# Functional role of skull lymphoid structures in CNS immunosurveillance

**DOI:** 10.1038/s41586-026-10951-4

**Published:** 2026-08-19

**Authors:** Jang Hyun Park, Daviti Abramishvili, Gustavo Gastão Davanzo, Ruben Silva, Xingxing Gu, Siling Du, Daniel D. Lee, Bernd H. Zinselmeyer, Jackson S. Turner, Gwendalyn J. Randolph, Igor Smirnov, Jonathan Kipnis

**Affiliations:** 1 Brain Immunology and Glia (BIG) Center, Washington University in St Louis, St Louis, MO USA; 2 Department of Pathology and Immunology, School of Medicine, Washington University in St Louis, St Louis, MO USA

**Keywords:** Neuroimmunology, Neuroimmunology

## Abstract

Accumulating evidence demonstrates that the central nervous system (CNS) is not disconnected from the peripheral immune system; however, precisely how the adaptive immune system surveils the CNS remains a critical question. Recent findings reveal that channels between the skull and the dura mater facilitate the exchange of cerebrospinal fluid and immune cells between the CNS and skull bone marrow of mice under both homeostatic and disease conditions^1–6^. Skull bone marrow functions as a source of immune cells for the CNS^5^, yet its role in CNS antigen-specific adaptive immune responses remains unclear. Here we identify lymphoid structures within the skull bone marrow, featuring germinal-centre-like formations and containing a distinct population of follicular-helper-like T cells that promote B cell activation and humoral immunity through CD40L, IL-21 and IFNγ signalling. Adaptive immune cells within these skull bone marrow lymphoid structures surveil and respond to CNS-derived antigens and contribute to anti-tumour immune responses in mouse brain cancer models. Together, our findings show that the skull bone marrow is a site of CNS immunosurveillance that may influence immune responses across diverse neurological diseases.

## Abstract (web summary)

Functional lymphoid structures within the skull bone marrow enable central nervous system immunosurveillance and shape immune responses to brain disease.

## Main

Recent findings have highlighted the direct connection between skull bone marrow (BM) and the dura mater through osseous channels, enabling cerebrospinal fluid (CSF) to reach the skull BM^1–4,7^. This connection is functionally important, as the skull BM is a primary lymphoid organ that supplies immune cells—including developing and immature B cells and myeloid cells—to the CNS and CNS border regions under homeostatic and neuroinflammatory or injurious conditions^5,6^. Recent studies also suggested that skull BM contains tumour-specific CD8^+^ T cells in human patients with glioblastoma and regulatory T cells in mouse tumour models^8,9^. Although the BM is a primary lymphoid organ, adaptive immune responses also occur within it under certain conditions^10–12^. Mature antigen-presenting cells (APCs), T cells and B cells migrate to the BM, where residing naive T and B cells can encounter antigens and form clusters^13–19^. While previous studies have shown that BM can support adaptive immune responses, whether and how skull BM contributes to CNS antigen recognition and immunosurveillance has not yet been elucidated.

Here we demonstrate that the skull BM of mice contains cellular components that are characteristic of peripheral lymphoid organs. APCs, T cells and B cells form clusters with germinal centre (GC)-like structures. These skull lymphoid structures participate in CNS immune surveillance under both homeostatic and brain cancer conditions in mice. By modulating their presence and activity in vivo, we show that these structures contribute to anti-tumour immunity.

## Existence of T_FH_ cells in the skull BM

**Extended Data Fig. 1:**
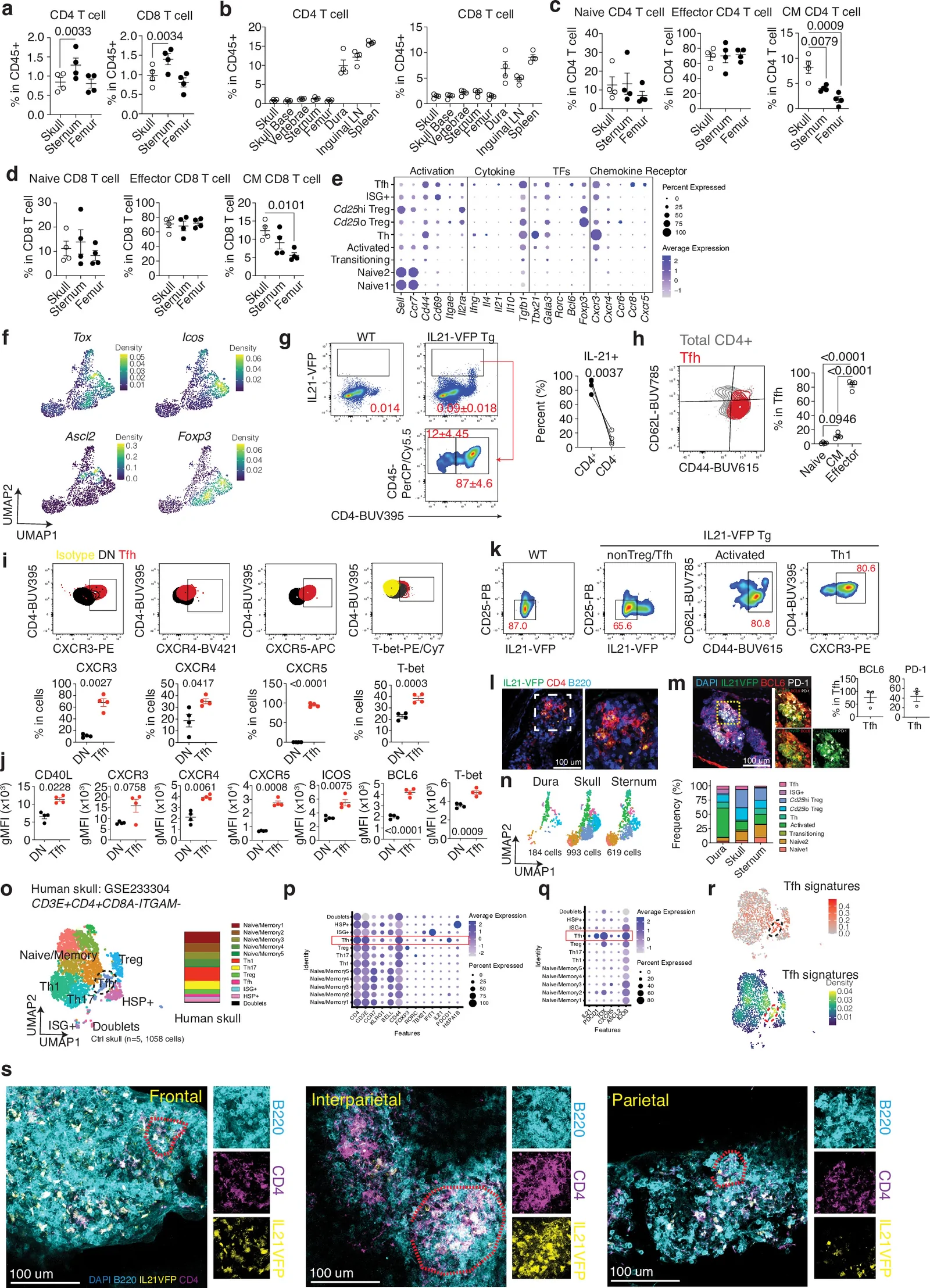
Characterization of Tfh cells. **a-d**. Flow cytometry analysis of T cells in different tissues: frequency of CD4 and CD8 T cells among CD45^+^ immune cells in skull, sternum, and femur (**a**) and additional tissues, including skull base, vertebrae, dura, inguinal LN, and spleen (**b**); frequency of naive, effector, and central memory (CM) subsets in CD4 (**c**) and CD8 (**d**) T cells. n = 4 of mice were used. **e**. Dot plots of indicated genes across clusters from scRNAseq analysis. **f**. Density plots for additional Tfh markers (*Tox, Icos, Ascl2*) and regulatory T cell marker (*Foxp3*). **g**. Flow cytometry analysis comparing the frequency of CD4^+^ and CD4^−^ cells among IL-21^+^ cells. n = 3 of mice were used. **h**. Flow cytometry analysis comparing the frequency of naive, CM, and effector stage in Tfh cells. n = 4 of mice were used. **i-j**. Flow cytometry analysis for marker expression percentage (**i**) and gMFI (**j**) in Tfh cells and IL-21^−^CXCR5^−^ DN cells from skull bone marrow. Representative FACS plot (top) and dot plots (bottom) are shown. n = 4 of mice. **k**. Flow cytometry analysis for defining Th1 marker expression of activated non-Treg/Tfh cells. **l-m**. Coronal sections of skull BM. **n**. Split UMAP plots by sample (top) and bar graphs showing the frequency of each cluster (bottom). **o-r**. Re-analysis of public scRNAseq data of human skull BM (GSE233304). UMAP visualization of clusters (above) and bar graph for percentage of each cluster (below) (**o**). Marker genes for cluster annotation (**p**) and Tfh marker genes (**q**). Feature and density plot for Tfh signature gene score (*IL21, PDCD1, TOX, CXCR5, ASCL2, ICOS*) (**r**). **s**. IHC images of the whole mount interparietal, parietal, and frontal skull. Data are represented as mean ± s.e.m. One-way ANOVA with Dunnett’s (**a, c, d**) or Tukey’s (**h**) multiple comparison, and two-tailed paired Student’s *t*-test (**g**, **i, j**) were used. *P* values are shown as the exact values. Source data

We compared T cell phenotypes in the skull BM to those of other BM sites in mice to characterize the adaptive immune composition of skull BM. The frequency of CD4^+^ and CD8^+^ T cells within total immune cells was similar across BM sites, with a higher frequency in the sternum BM, but overall lower than in secondary lymphoid organs (SLOs) (Extended Data Fig. 1a,b). The frequencies of naive and effector T cells were also comparable. However, skull BM displayed a higher proportion of central memory cells compared with other BM sites (Extended Data Fig. 1c,d), suggesting a distinct T cell environment within the skull BM.

**Fig. 1:**
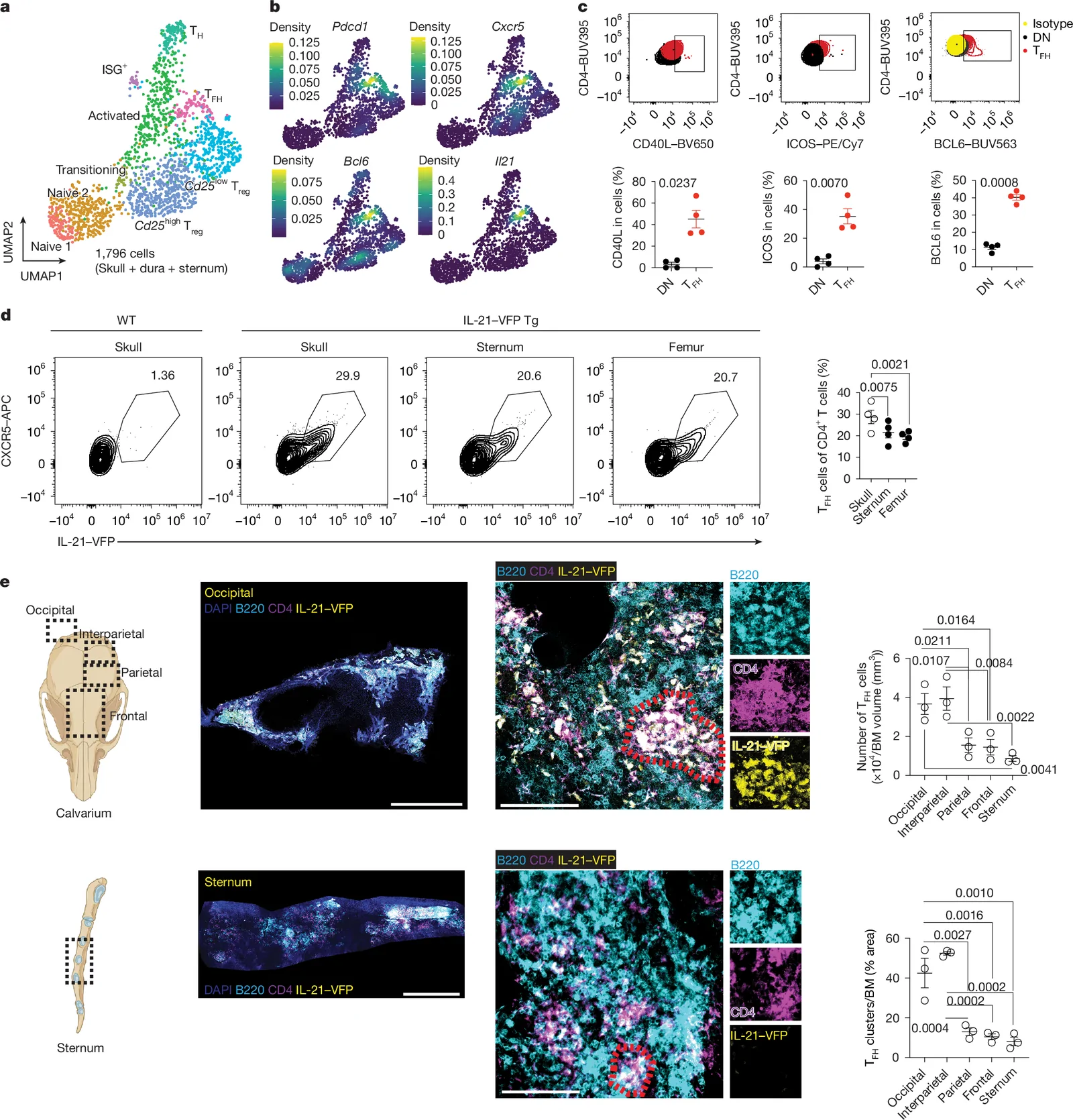
Identification of BM T_FH_ cells. **a**, Uniform manifold approximation and projection (UMAP) visualization of scRNA-seq data for CD4^+^ T cells from skull, sternum BM and dura, pooled from *n* = 20 mice, based on marker gene expression. ISG^+^, ISG^+^ cells; activated, activated T cells; transitioning, transitioning cells; naive 1, 2, naive T cells. **b**, Expression density plots of the indicated genes. **c**, Flow cytometry analysis of marker expression in T_FH_ cells (live, singlet, CD45^+^CD3^+^CD4^+^IL-21–VFP^+^CXCR5^+^) and IL-21^−^CXCR5^−^ double-negative (DN) cells (live, singlet, CD45^+^CD3^+^CD4^+^IL-21–VFP^−^CXCR5^−^) from skull BM. Representative fluorescence-activated cell sorting (FACS) plots (top) and dot plots (bottom) are shown. *n* = 4 mice. **d**, Flow cytometry data for IL-21–VFP^+^CXCR5^+^ T_FH_ cells from the skull, sternum and femur BM of WT and IL-21–VFP transgenic (Tg) mice. Representative plots (left) and dot plots (right) are shown. *n* = 4 mice. **e**, Whole-mount immunohistochemistry (IHC) image of the occipital calvarium and sternum, along with quantification. *n* = 3 mice. Data are mean ± s.e.m. Statistical analysis was performed using a two-tailed paired Student’s *t*-tests (**c**) and one-way analysis of variance (ANOVA) with Dunnett’s (**d**) or Tukey’s (**e**) multiple-comparison test. Exact *P* values are shown. The diagram in **e** was created using BioRender; Kipnis, J. https://biorender.com/icon/mouse-skull-dorsal (skull), https://biorender.com/icon/sternum-lateral (sternum) (2026). For **e**, scale bars, 2 mm (top left), 500 μm (bottom left) and 100 μm (right). Source data

To further examine T cell phenotypes in BM, we conducted 10x single-cell RNA sequencing (scRNA-seq) analysis of T cells from mouse skull and sternum BMs. On the basis of clustering, we identified diverse CD4^+^ T cell subsets, including naive (*Sell* and* Ccr7*), activated (*Cd44*), interferon-stimulated gene (ISG)-expressing (*Isg15*), regulatory (*Foxp3*) and differentiated helper (*Tbx21*, *Gata3* and* Rorc*) cells (Fig. 1a and Extended Data Fig. 1e). Notably, we observed follicular helper T (T_FH_) cells marked by the expression of genes such as *Pdcd1*,* Cxcr5*,* Bcl6* and *Il21* (ref. ^20^) (Fig. 1a,b, and Extended Data Fig. 1e,f).

To validate these findings at the protein level, we performed flow cytometry analysis of BM from IL-21–VFP (vivid Verde fluorescent protein) mice, and identified IL-21^+^CXCR5^+^ T_FH_ cells as a distinct subset (Fig. 1d). We confirmed that CD4^+^ T cells are the primary source of IL-21 in the BM (Extended Data Fig. 1g), with the majority of T_FH_ cells in an effector stage and some in a central memory stage (Extended Data Fig. 1h). Protein-level expression of markers on T_FH_ cells was higher than on IL-21^−^CXCR5^−^ double-negative cells (Fig. 1c and Extended Data Fig. 1i,j). These T_FH_ cells also expressed T helper type 1 (T_H_1) markers, such as CXCR3 and T-bet, and the majority of activated non-regulatory T (T_reg_) cells and non-T_FH_ cells were T_H_1 cells (Extended Data Fig. 1i–k). The presence of T_FH_ cells in the skull BM was also confirmed by confocal microscopy (Extended Data Fig. 1l,m).

Skull BM exhibited a higher percentage of T_FH_ cells than other BM sites (Fig. 1d and Extended Data Fig. 1n). Analysis of public scRNA-seq data from human skull BM (GSE233304)^8^ also supported the presence of T_FH_-like cells, despite their low abundance (Extended Data Fig. 1o–r). Confocal imaging analysis of whole-mounted calvarium demonstrated that T_FH_ cells form clusters with B cells in the BM of occipital and interparietal regions of the skull (Fig. 1e and Extended Data Fig. 1s). However, these cells were rare in the BMs of parietal and frontal skull bones as well as in sternum BM.

In summary, T_FH_ cells are present in the BMs of various bones, and skull BM contains a higher proportion of these cells compared with other sites, with preferential forming of clusters with B cells under homeostatic conditions.

## Activation of B cells in skull BM

**Extended Data Fig. 2:**
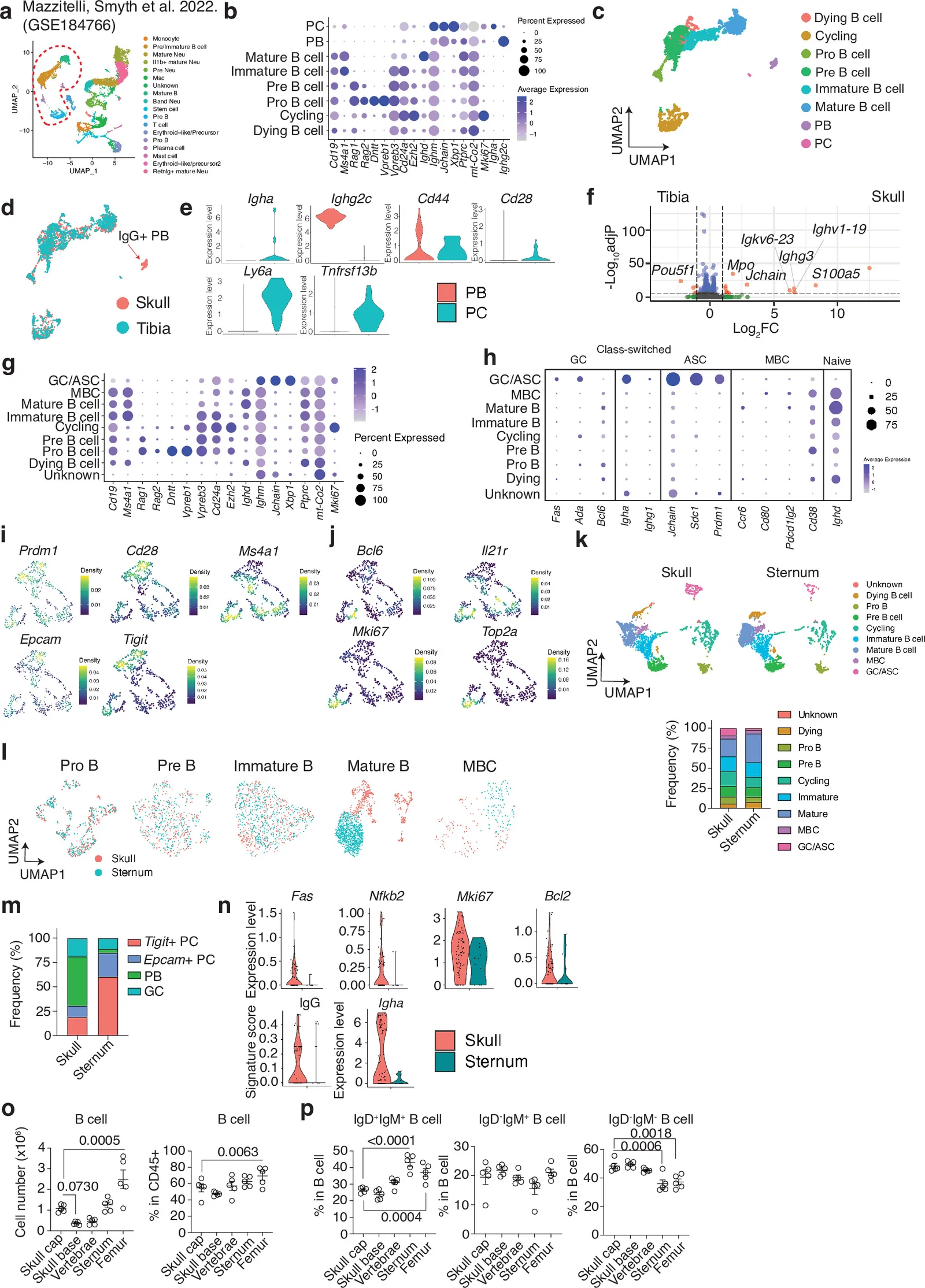
Skull BM provides a favourable environment for B cell activation and differentiation. **a**. Re-analysis of scRNAseq public data (GSE184766). UMAP visualization of clusters; B-linage cells highlighted (red circle). **b**. Dot plot of marker genes for B-lineage cells. **c**. Re-clustering of B-lineage cells. **d**. Overlapped UMAP plot grouped by batch; red arrow indicates IgG^+^ plasmablast (PB). **e**. Violin plots of marker genes; Immunoglobulins (*Igha, Ighg2c*), PB marker (*Cd44*), and plasma cell marker (*Cd28, Ly6a, Tnfrsf13b*). **f**. Volcano plot of differentially expressed genes (DEGs) from whole-cell populations of each tissue. **g-h**. Dot plots of marker genes for lineage identification (**g**) and activation/differentiation-related genes (**h**). **i**. Density plots showing the expression of the ASC marker (*Prdm1*), plasma cell markers (*Cd28, Epcam, Tigit*), and the plasmablast marker (*Ms4a1*). **j**. Density plots for the expression of GC markers. **k**. Split UMAP plots by batch (top) and bar graphs showing cluster frequency (bottom). **l**. Overlapped UMAP plots of clusters grouped by batch. **m**. Bar graph showing frequency of each cluster from Fig. 2d. **n**. Violin plots displaying the expression of indicated genes: GC markers (*Fas, Nfkb2, Mki67, Bcl2*), IgG signature (*Ighg1, Ighg2c, Ighg2b, Ighg3*), and *Igha*. **o**. Flow cytometry analysis of B cells; cell number and frequency among CD45^+^ immune cells. **p**. Subset composition of B cells. Data are represented as mean ± s.e.m. n = 5 of mice were used. One-way ANOVA with Dunnett’s multiple test was used. *P* values are shown as the exact values. Source data

On the basis of the observation of a higher number of T_FH_ cells in the skull BM, we hypothesized that the skull BM might exhibit stronger B cell responses compared with other BM sites. Analyses of an available scRNA-seq dataset (GSE184766)^4^ identified a skull-BM-specific cluster that was absent in the tibia BM (Extended Data Fig. 2a–d). While both BMs contained IgA-expressing plasma cells (PCs), the skull BM contained a distinct population of IgG^+^ plasmablasts (PBs) (Extended Data Fig. 2c–e). A pseudobulk comparison of differentially expressed genes between skull and tibia BM immune cells revealed that most differentially expressed genes in skull BM immune cells were related to antibody responses (such as *Jchain* and* Ighg3*) (Extended Data Fig. 2f), suggesting that the skull BM may provide a favourable environment for B cell activity.

**Fig. 2:**
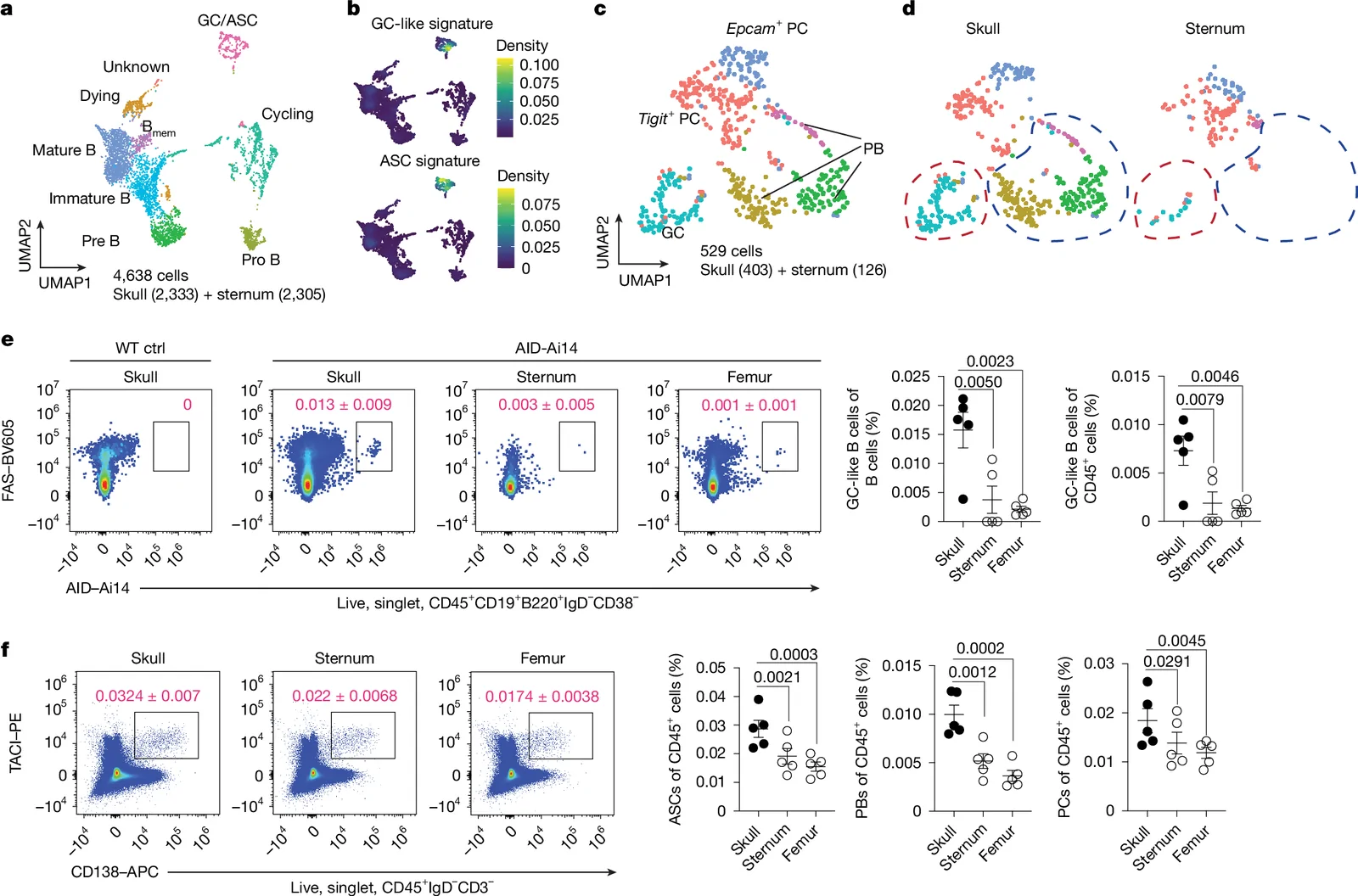
Skull BM B cells are consistently activated under homeostasis. **a**, UMAP visualization of scRNA-seq data for B cells and ASCs from skull and sternum BM, pooled from *n* = 20 mice, based on marker gene expression. GC/ASC, GCs and ASCs; B_mem_, memory B cells; pre B, pre-B cells; pro-B, pro-B cells. **b**, The expression of GC-like signature genes (*Fas*,* Ada* and *Bcl6*) and ASC signature genes (*Jchain*,* Sdc1* and* Prdm1*). **c**, UMAP plot of the reclustered GC/ASC cluster. **d**, Split UMAP plots of the reclustered GC/ASC cluster, showing the GC-like cluster (red circle) and PBs (blue circle). **e**, Flow cytometry analysis of GC-like B cells from skull, sternum and femur BM using AID-Ai14 reporter mice. Representative plot (left) and dot plots (right) are shown. *n* = 5 mice. **f**, Flow cytometry analysis of ASCs, PBs and PCs from skull, sternum and femur BM. Representative plots (left) and dot plots (right) are shown.* n* = 5 mice. Data are mean ± s.e.m. Statistical analysis was performed using one-way ANOVA with Dunnett’s multiple-comparison test. Exact *P* values are shown. Source data

We next performed scRNA-seq analysis of isolated B cells and antibody-secreting cells (ASCs) from mouse skull and sternum BM. Most B cells were in early developmental stages (Fig. 2a and Extended Data Fig. 2g). Notably, there was a cluster of B cells expressing GC-related signatures (for example, *Fas*,* Ada* and* Bcl6*) and ASC signatures (such as *Jchain*,* Sdc1* and* Prdm1*) (Fig. 2b and Extended Data Fig. 2h). These cells expressed class-switched immunoglobulins but lacked memory markers and *Ighd* expression. Reclustering this group, we identified four subsets based on marker expression: *Epcam*^+^ PCs, *Tigit*^+^ PCs, PBs and GC-like B cells, which expressed genes associated with GCs, such as *Bcl6*,* Il21r*,* Mki67* and *Top2a* (Fig. 2c and Extended Data Fig. 2i,j). The skull BM had a higher proportion of cells in the GC and ASC (GC/ASC) cluster than the sternum BM (Extended Data Fig. 2k).

While overall B cells, including developing B cells, exhibited similar transcriptional patterns across BMs, mature B cells, memory B cells and GC/ASC clusters showed distinct profiles between skull and sternum BM (Fig. 2d and Extended Data Fig. 2l). Consistent with previous results, the skull BM contained a higher proportion of PBs (Fig. 2d). Although GC-like B cells were present in the sternum BM, they were more abundant in the skull BM, where they exhibited higher GC marker expression, suggesting closer resemblance to GCs (Fig. 2d and Extended Data Fig. 2m,n).

We confirmed these observations at the protein level. While the total B cell frequency among immune cells was similar across BMs, the skull BM had a higher percentage of IgD^–^IgM^–^ developing B cells or class-switched cells and fewer IgD^+^IgM^+^ mature B cells than the sternum and femur BMs (Extended Data Fig. 2o,p). Using both protein markers (IgD^–^CD38^–^FAS^+^) and activation-induced cytidine deaminase (AID)-Ai14 reporter mice, we confirmed the presence of GC-like B cells, which were more abundant in the skull BM than in other BM sites, while the frequency of class-switched memory B cells was comparable (Fig. 2e and Supplementary Fig. 1a–d). Similarly, the skull BM contained more ASCs than other BM sites (Fig. 2f and Supplementary Fig. 1e–h).

In conclusion, the skull BM exhibits a higher activation status and a greater abundance of GC-like B cells and ASCs compared with other BMs.

## T_FH_ cells promote B cells in skull BM

Canonical B cell activation and differentiation typically require CD4^+^ T cell-derived cytokines, such as IL-21, and co-stimulatory molecules, such as CD40L^21^. To assess whether T_FH_ cells support canonical B cell activation and differentiation in the skull BM, we first depleted CD4^+^ T cells (or CD8^+^ T cells) using antibodies (Supplementary Fig. 2a,b). CD4^+^ T cell depletion resulted in a significant reduction in ASC differentiation in the skull BM; CD8^+^ T cell depletion had no significant effect on ASC differentiation (Supplementary Fig. 2c). As the cytokine IL-21 is crucial for early B cell activation and is commonly produced by T_FH_ cells^22^, we examined skull BM of IL-21 receptor (*Il21r*)-deficient mice and found that skull GC-like B cells and ASCs were dependent on IL-21 signalling, similar to splenic cells (Supplementary Fig. 2d–g). However, total B cells and other immune cells in the skull BM were unaffected by deletion of *Il21r* (Supplementary Fig. 2h). Moreover, we showed that skull GC-like B cells rely on the CD40–CD40L axis, as systemic CD40L neutralization using anti-CD40L antibodies reduced GC-like B cells without affecting the total T or B cell numbers (Supplementary Fig. 3a–c).

To assess the local requirement of T–B cell interactions within the skull BM, we delivered a hydrogel containing anti-CD40L antibody subcutaneously (s.c.) under the scalp. Delivered antibodies preferentially bound to T_FH_ cells in the skull BM, but were under detection threshold in other tissues, supporting a relatively localized effect (Supplementary Fig. 3d). This treatment reduced the number of GC-like B cells and ASCs specifically in the skull BM, while these cells were unaffected in the sternum BM, dura and spleen (Supplementary Fig. 3e–j). On the other hand, peripheral hydrogel delivery of anti-CD40L into the flank did not affect GC-like B cells in the skull BM, whereas draining inguinal lymph nodes (LNs) showed depletion (Supplementary Fig. 3k). Together, these findings suggest that T_FH_ cells support B cell activation and differentiation within the skull BM.

## Lymphoid structures in the skull BM

**Fig. 3:**
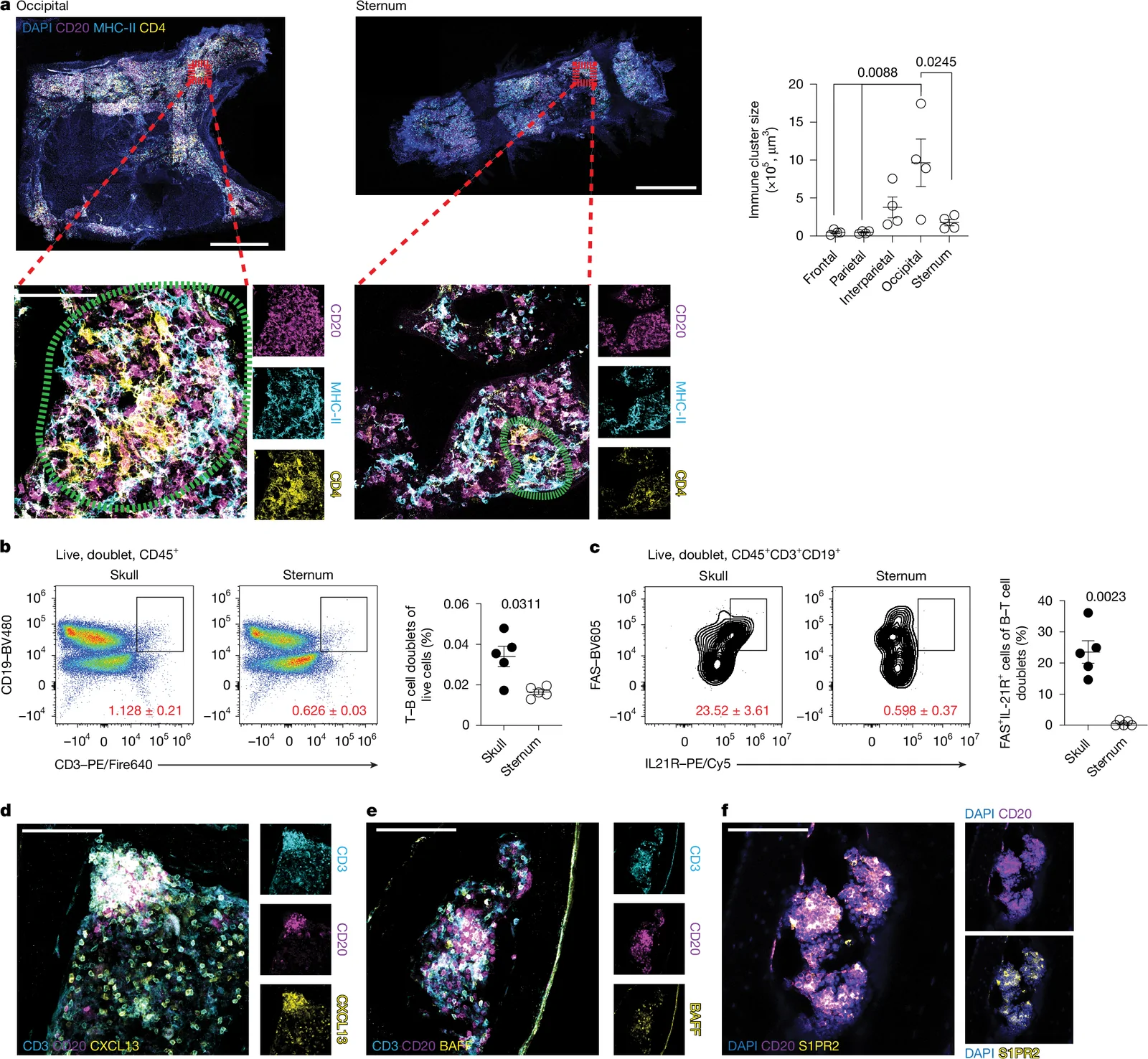
Skull BM contains lymphoid clusters. **a**, Whole-mount IHC images of the occipital skull and sternum BM (left), along with quantification (right). *n* = 4 mice. Scale bars, 1 mm (top) and 100 μm (bottom). **b**, Flow cytometry analysis of T–B cell doublets. *n* = 5 mice. **c**, Flow cytometry analysis of FAS- and IL-21R-expressing T–B cell doublets. Representative plots (left) and dot plots (right) are shown. *n* = 5 mice. **d**, IHC image of a coronal section of skull BM to visualize CXCL13 expression in B cell aggregates. **e**, IHC image of a coronal section of skull BM to visualize BAFF expression in B cell aggregates. **f**, IHC image of a coronal section of skull BM to visualize S1PR2-expressing GC B cells. Data are mean ± s.e.m. Statistical analysis was performed using one-way ANOVA with Tukey’s multiple-comparison test (**a**) and two-tailed paired Student’s *t*-tests (**b** and** c**). Exact *P* values are shown. For **d**–**f**, scale bars, 100 μm. Source data

**Extended Data Fig. 3:**
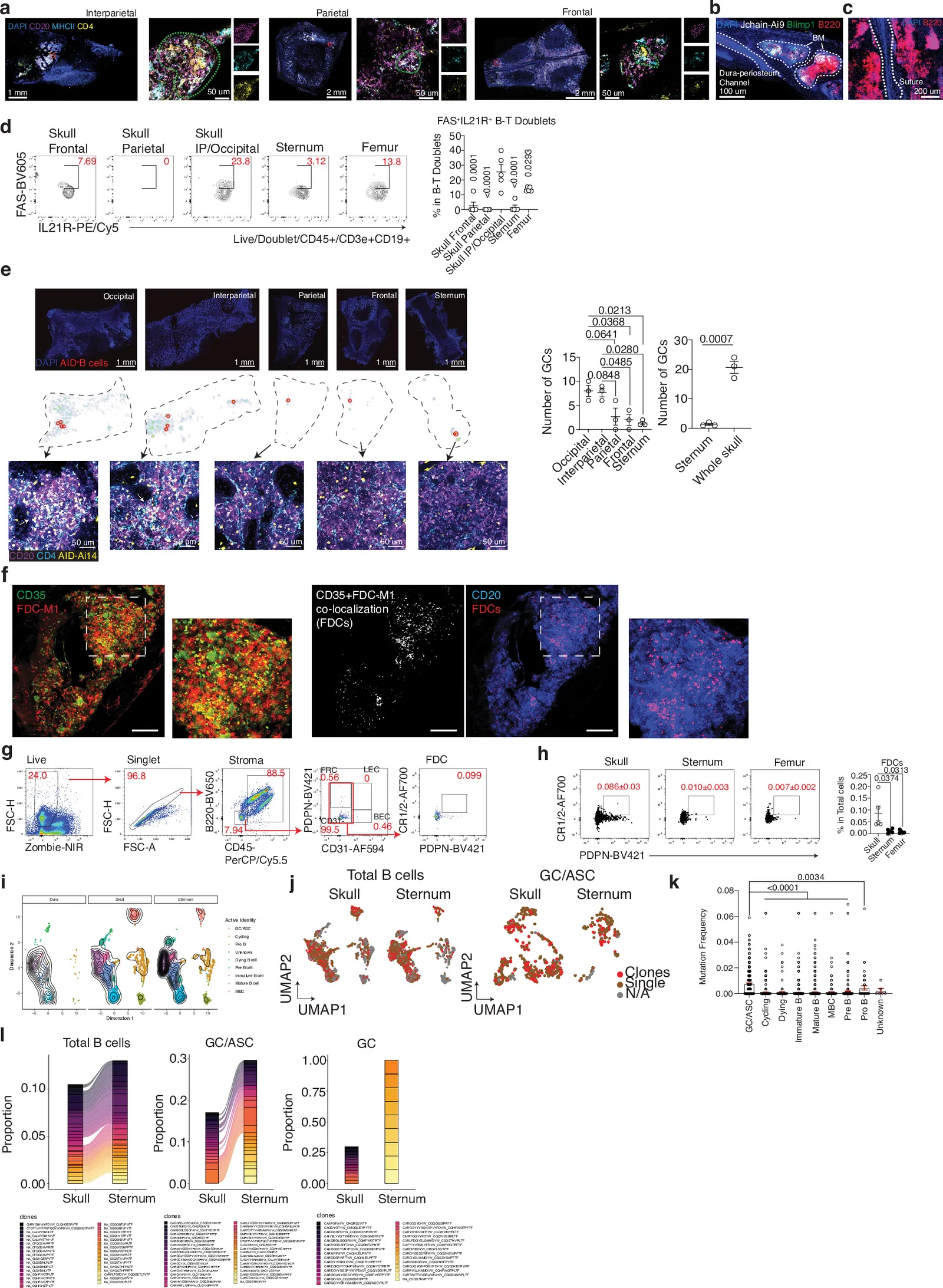
Additional evidence of GC-like structures in skull BM. **a**. Whole mount IHC image of the interparietal, parietal, and frontal skull BM. Related to Fig. 3a. **b**. Sagittal skull section of Jchain-CreERT2::Ai9 mice. **c**. Whole mount skull showing axial view of suture. **d**. FAS^+^IL21R^+^ GC doublets in each bone (representative FACS plot, left; quantification, right). n = 5 of mice were used. **e**. Germinal centres quantification: whole mount bones (upper), density maps of AID^+^CD20^+^ double positive cells (middle), representative zoomed images (bottom) and quantification (right). n = 3 of mice per group were used. **f**. Follicular dendritic cell (FDC) marker expression in skull BM coronal sections: CD35^+^ and FDC-M1^+^ FDCs (left), co-localization with CD20^+^ B cell aggregates (right). Selected areas shown at right. Scale bar, 100 μm. These are representative images for three independent experiments. **g**. Gating strategy for FDCs. **h**. Frequency of FDCs among live cells per each tissue. n = 5 of mice were used. **i**. BCR clonal expansion contour plots from scRNAseq clusters. **j**. UMAP plots of total B cells (left) and GC/ASC clusters (right) showing clonal expansion (single, n = 1 of BCR clone; clones, n > =2 of BCR clones). **k**. IGHV mutation frequency by each identity. Cell numbers: ASC/GC = 457, Cycling = 582, Dying = 183, Immature B = 907, Mature B = 1200, MBC = 218, Pre B = 365, Pro B = 46, Unknown = 6. **l**. BCR repertoire proportions and shared clones between skull and sternum BM. Data are represented as mean ± s.e.m. Two-tailed paired Student’s *t*-test (**e**) and One-way ANOVA with Tukey’s (**e**) or Dunnett’s (**d, h, k**) multiple comparison was used. *P* values are shown as the exact values. Source data

We observed that IL-21-expressing T_FH_ cells form clusters with B cells in the skull BM (Fig. 1e), suggesting the presence of lymphoid structures^20^. To investigate further, we performed confocal imaging of cleared mouse calvarium to examine clustering among MHC-II^+^ APCs, CD4^+^ T cells and CD20^+^ B cells. B cells formed clusters with APCs and T cells predominantly in the posterior regions of the skull BM, such as the occipital and interparietal areas of the calvaria bone, compared with other areas of the skull BM and sternum BM (Fig. 3a and Extended Data Fig. 3a). However, such immune cell organization was not observed in non-marrow regions, such as the diploic vein channels and sutures (Extended Data Fig. 3b,c).

A doublet assay also confirmed that the skull BM had more frequent T–B cell interactions than the sternum BM (Fig. 3b), with skull doublets expressing FAS and IL-21R, markers that are absent in doublets from the sternum and anterior skull BM (Fig. 3c and Extended Data Fig. 3d). Femur BM exhibited substantial amounts of FAS^+^IL-21R^+^ doublets; however, the percentage was lower than in the posterior skull BM. These findings indicate that the skull BM hosts closely interacting T and B cells, forming GC-like clusters in the presence of APCs, which are more common in the posterior than anterior skull BM.

To further determine whether these structures exhibited GC-like characteristics, we investigated multiple GC components. First, the expression of the B cell chemoattractant CXCL13 and B cell activating factor (BAFF) was co-localized within these clusters (Fig. 3d,e); second, we observed S1PR2-expressing B cell clusters (Fig. 3f); third, we used AID-Ai14 reporter mice to confirm GC-like structures (Extended Data Fig. 3e); and finally, we observed follicular dendritic cells (FDCs) within the B cell-rich area (Extended Data Fig. 3f), and flow cytometry confirmed that the skull BM contained FDCs while other BM sites did not (Extended Data Fig. 3g,h).

Moreover, single-cell BCR-sequencing analysis revealed that GC-like B cells and ASCs displayed clonal expansion and evidence of somatic hypermutation (Extended Data Fig. 3i–k). Some BCR clones were shared between the skull and sternum BM, skull-specific clones of GC-like B cells and ASCs were also identified (Extended Data Fig. 3l).

**Extended Data Fig. 4:**
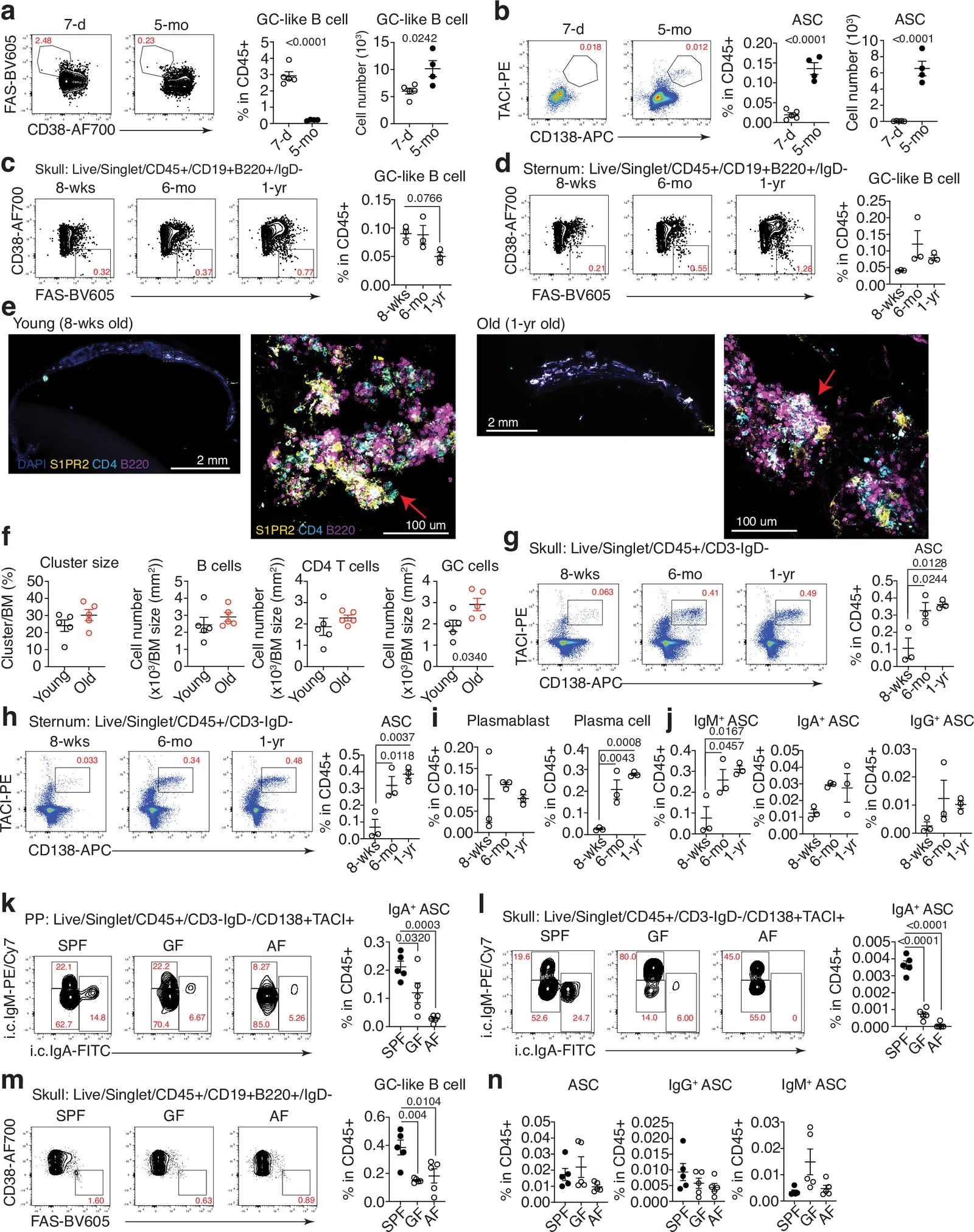
Impact of commensal microbiota and ageing on skull BM B cell activation. **a-b**. Number and frequency of GC-like B cells (**a**) and ASCs (**b**) among total immune cells in skull BM of 7-day-old versus 5-month-old mice. n = 5 (7-d) and n = 4 (5-mo) of mice were used. **c-d**. Frequency of GC-like B cells among total immune cells in skull (**c**) and sternum (**d**) from mice of different ages. n = 3 mice per group were used. **e-f**. Coronal posterior skull sections from young and old mice (images, **e**; quantification, **f**). n = 5 of mice per group were used. **g-h**. Frequency of ASCs among total immune cells in skull (**g**) and sternum BM (**h**) with age. n = 3 of mice were used. **i-j**. Frequency of plasmablasts, plasma cells (**i**), and immunoglobulin-expressing ASC subsets (**j**). n = 3 of mice were used. **k-n**. Frequency of IgA^+^ ASCs in Peyer’s patch (PP) (**k**) and skull BM (**l**), GC-like B cells in skull BM (**m**), and ASCs plus ASC subsets in skull BM (**n**) from specific pathogen free (SPF), germ free (GF), and antigen free (AF) mice. n = 5 of mice per group were used. Two-tailed unpaired Student’s *t-*test (**a, b, f**) and one-way ANOVA with Tukey’s (**c, g-j**) or Dunnett’s (**k-n**) multiple tests were used. Data are represented as mean ± s.e.m. *P* values are shown as the exact values. Source data

Notably, GC-like B cells were already present at 7 days postnatal, with substantial numbers, although ASCs were significantly less abundant at this early stage (Extended Data Fig. 4a,b). In 1-year-old mice, the percentage of overall GC-like B cells did not increase, while the posterior skull section exhibited a slight increase in S1PR2^+^ GC cells (Extended Data Fig. 4c–f) and ASCs increased in both the skull and sternum BM (Extended Data Fig. 4g–j).

In summary, the skull BM contains lymphoid structures with GC-like features that are established from the early postnatal period and into ageing.

## Antigen-specific responses in skull BM

Under homeostasis, lymphoid structures in various organs may respond to self-antigens from tissues and antigens from microbiota and diet^23^. To investigate the antigens that activate cells within the skull lymphoid structures, we compared specific pathogen-free (SPF) and germ-free (GF) mice, and GF mice on an amino-acid-based antigen-free (AF) diet. As expected, IgA^+^ ASCs in Peyer’s patches were reduced in the GF and AF mice compared with in the SPF mice (Extended Data Fig. 4k). Similarly, IgA^+^ ASCs in the BM are also gut microbiota dependent^24^. Consistently, IgA^+^ ASCs and GC-like B cells in the skull BM were decreased in GF and AF mice compared with in SPF mice (Extended Data Fig. 4l,m). However, a fraction of GC-like B cells remained, and IgG^+^ and IgM^+^ ASCs were not affected across groups (Extended Data Fig. 4m,n). Given that GC-like B cells and ASCs in the skull BM underwent somatic hypermutation and clonal expansion (Extended Data Fig. 3i–k), we hypothesized that the remaining GC-like B cells and ASCs in GF and AF mice are responding to self-antigens^25–28^.

**Extended Data Fig. 5:**
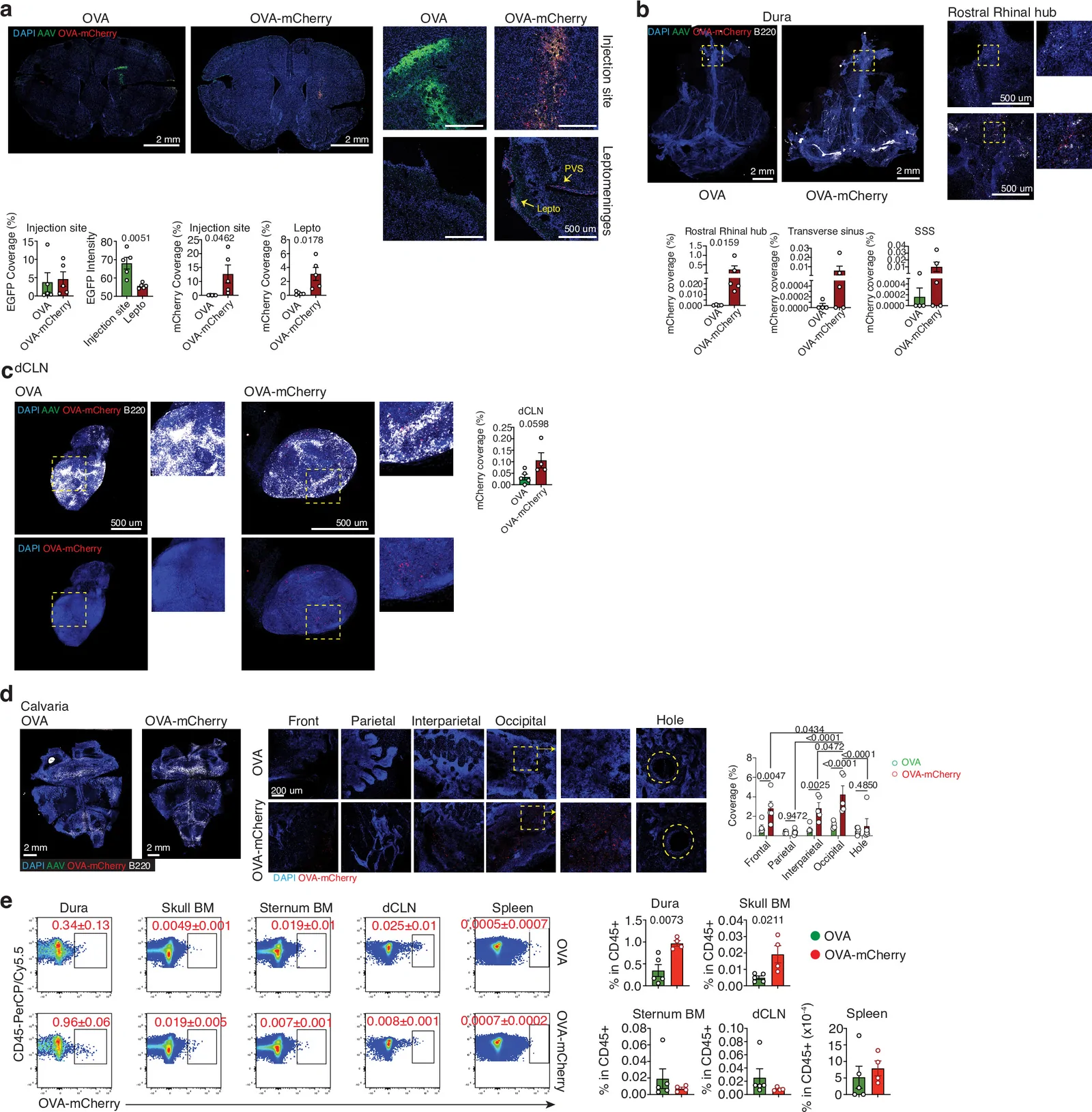
Validation of AAV-mediated antigen delivery system. **a-d**. After 4 weeks of AAV-hSyn-OVA-P2A-EGFP (OVA) or AAV-hSyn-OVA-mCherry-P2A-EGFP (OVA-mCherry) injection, IHC analysis was conducted. Confocal images of coronal brain sections with whole brain (upper left), zoomed in images of injection site and leptomeninges (right), and quantifications (bottom) (**a**). n = 5 of mice per group were used. Whole mount dura (left) with zoomed in images of rostral rhinal hubs (right), and quantifications (bottom) (**b**). n = 4 (OVA) and n = 5 (OVA-mCherry) mice were used. Tissue-cleared whole mount dCLNs (left) with quantifications (right) (**c**). n = 5 (OVA) and n = 4 (OVA-mCherry) mice were used. Tissue-cleared whole mount skull images (left), zoomed in images of specific regions (middle), and quantifications (right) (**d**). n = 5 mice per group were used. **e**. Flow cytometry analysis of each tissue after 4 weeks of AAV injections. n = 5 (OVA) and n = 4 (OVA-mCherry) mice were used. Data are represented as mean ± s.e.m. Two-tailed unpaired Student’s *t*-test (**a, c, e**), Two-tailed unpaired Mann-Whitney test (**b**), and two-way ANOVA with Tukey’s (**d**) were used. *P* values are shown as the exact values. Source data

To test this, we used an adeno-associated virus (AAV) delivery system with a human synapsin 1 (hSyn) promoter to induce the expression of model antigens in neurons. We used AAV-hSyn-OVA-mCherry-P2A-eGFP, with AAV-hSyn-OVA-P2A-eGFP as a control. In this system, eGFP is retained in neurons, whereas ovalbumin (OVA)-conjugated mCherry can be detected on release. AAV-mediated gene expression was confirmed by eGFP and/or mCherry signals in the brain 1 month after infection. OVA–mCherry was detected not only at the injection site but also in the perivascular spaces and leptomeninges, whereas eGFP signals remained restricted to neurons. Importantly, mCherry signals were not detected leaking through the injection tract (Extended Data Fig. 5a).

OVA–mCherry was detected as expected^4,27,29^ in the rostral rhinal hub of the dura (Extended Data Fig. 5b), deep cervical LNs (dCLNs) (Extended Data Fig. 5c) and the skull BM, where occipital BM showed the strongest signal, whereas the burr hole was negative (Extended Data Fig. 5d). Similarly, flow cytometry also revealed OVA–mCherry^+^ immune cells in the dura and skull BM, but not in other tissues (Extended Data Fig. 5e). Tracer experiments confirmed that craniotomy injury does not make an artificial conduit for CSF, and the absence of AAV-derived *eGFP* mRNA in the skull BM and dura excluded leakage of AAVs from craniotomy surgery (Supplementary Fig. 4).

Together, these data demonstrate that intracranial AAV delivery does not induce artificial antigen leakage and that antigens reach brain border tissues, probably through known physiological routes.

**Fig. 4:**
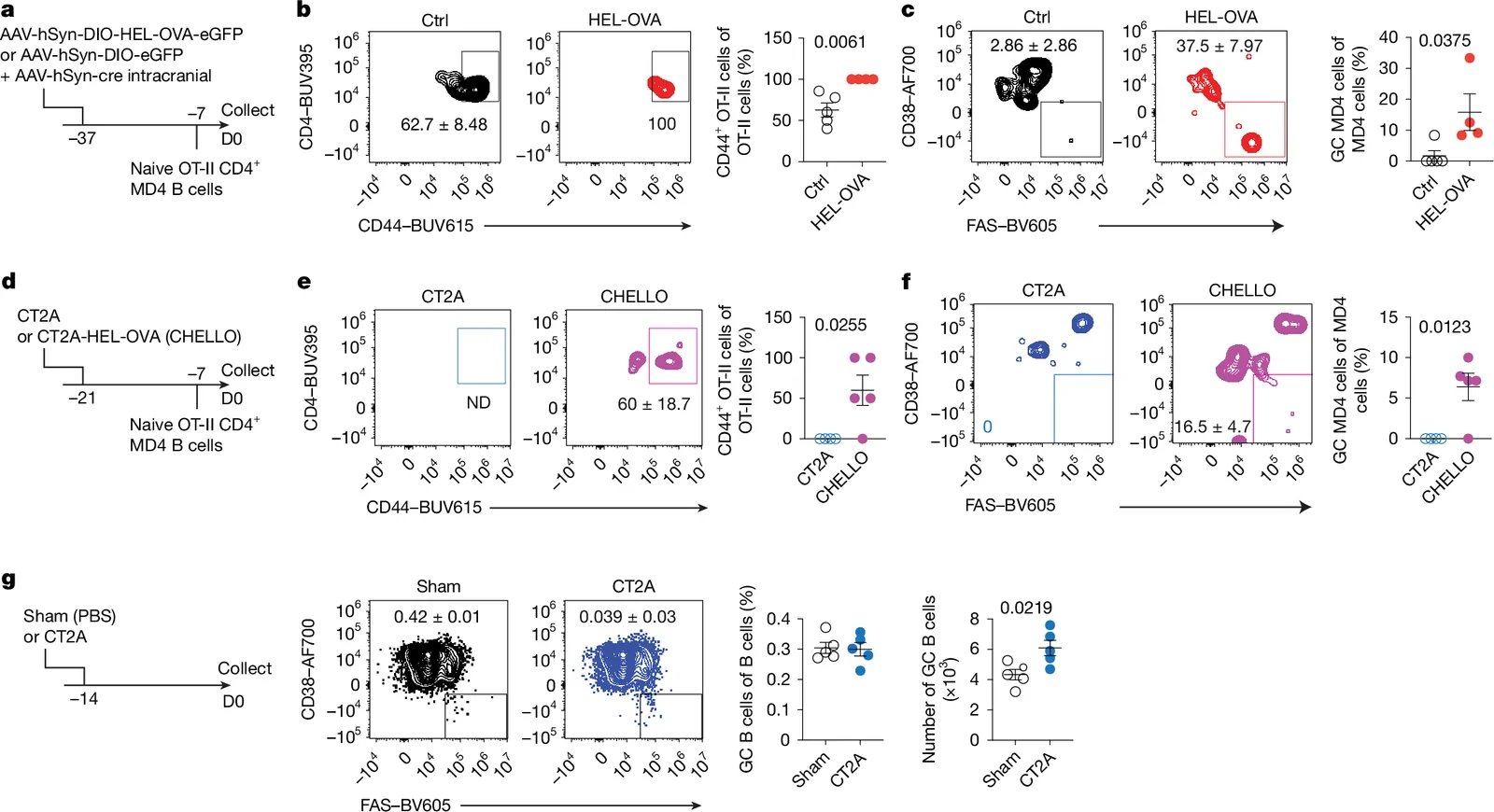
Skull lymphoid structures respond to brain-derived antigens. **a**, Experimental scheme for AAV injection. D0, day 0; DIO, double-floxed inversed ORF. **b**,**c**, Flow cytometry analysis of the frequency of CD44^+^ activated cells in OT-II CD4^+^ T cells (**b**) and GC-like cells in MD4 B cells (**c**) in the skull BM. *n* = 5 (ctrl) and *n* = 4 (HEL-OVA) mice. **d**, Experimental schematic of tumour injection. **e**,**f**, Flow cytometry analysis of the frequency of CD44^+^ activated cells in OT-II CD4^+^ T cells (**e**) and GC-like cells in MD4 B cells (**f**) in the skull BM. *n* = 4 (CT2A) and *n* = 5 (CHELLO) mice. **g**, Experimental schematic (left) and flow cytometry analysis (right) of the frequency and number of GC-like B cells in the skull BM.* n* = 5 mice. Data are mean ± s.e.m. Statistical analysis was performed using the two-tailed unpaired Student’s *t*-test. Exact *P* values are shown. ND, not detected. Source data

**Extended Data Fig. 6:**
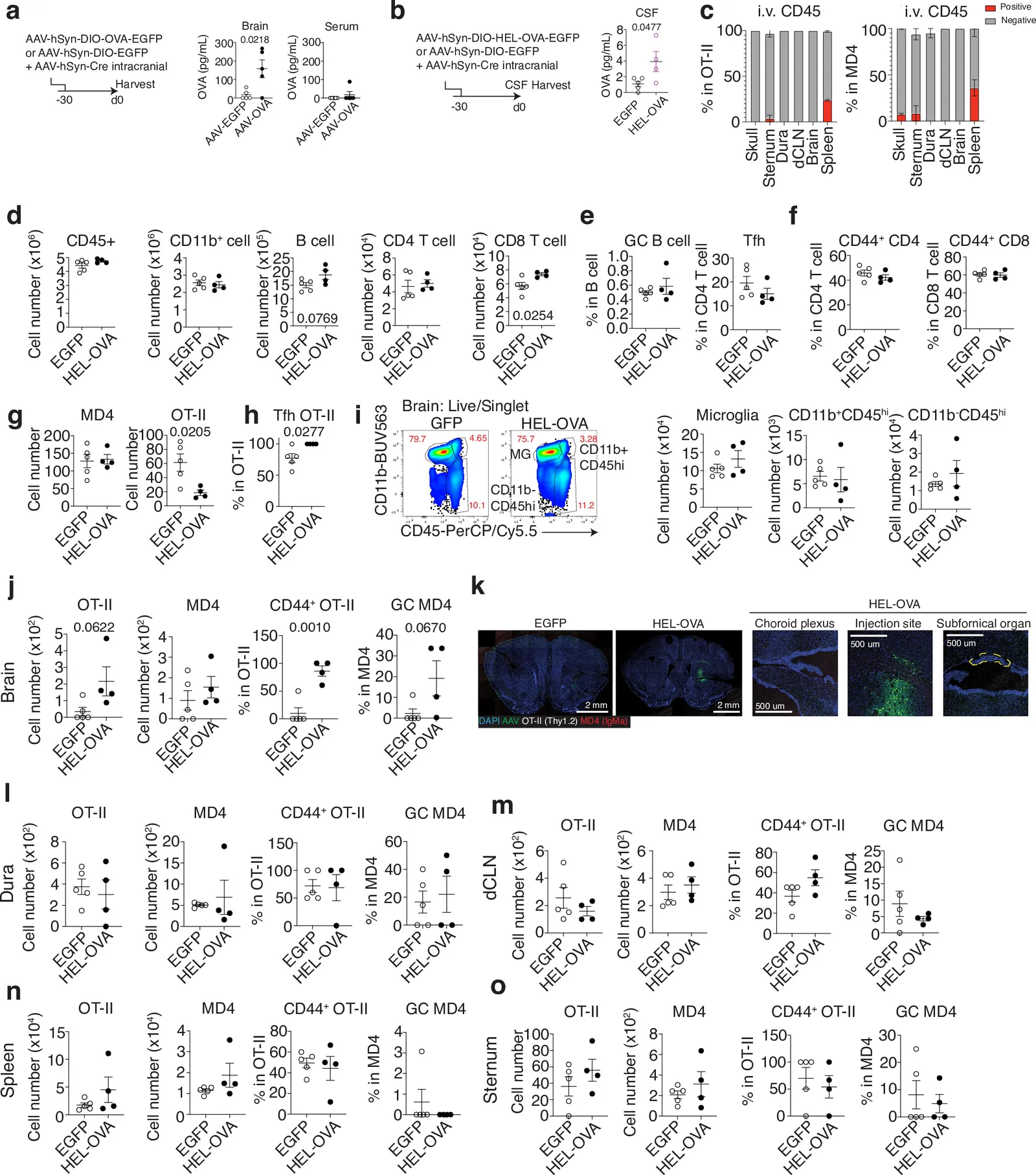
The effect of AAV-HEL-OVA for systemic immune responses. **a**. AAV injection scheme (left); OVA levels in brain and serum (right). n = 5 of mice per group were used. **b**. OVA levels in CSF (right) with experimental scheme (left). n = 5 (EGFP) or n = 4 (HEL-OVA) mice were used. **c**. Bar graphs for intravenously (i.v.) CD45-labelled OT-II (left) and MD4 (right) cells in each tissue. n = 3 of mice were used. **d-h**. One month post AAV-EGFP or AAV-HEL-OVA, OT-II and MD4 cells were adoptively transferred; one week later, skull BM immune cells were analysed; immune cell numbers (**d**); frequencies of differentiated B and T cells (**e**); activated CD4 and CD8 T cells (**f**); number of MD4 B cells and OT-II CD4 T cells (**g**); frequency of Tfh cells among OT-II CD4 T cells (**h**). n = 5 (EGFP) and n = 4 (HEL-OVA) mice were used. **i**. Whole brain flow cytometry; number of microglia, CD11b^−^CD45^hi^, and CD11b^+^CD45^hi^ cells. n = 5 (EGFP) and n = 4 (HEL-OVA) mice were used. **j**. OT-II and MD4 responses in brain. n = 5 (EGFP) and n = 4 (HEL-OVA) of mice were used. **k**. IHC images of coronal brain sections after AAV-HEL-OVA injection and OT-II/MD4 co-transfer. **l-o**. OT-II and MD4 responses in dura (**l**), dCLN (**m**), spleen (**n**), and sternum BM (**o**). n = 5 (EGFP) and n = 4 (HEL-OVA) of mice were used. Data are represented as mean ± s.e.m. Two-tailed unpaired Student’s *t-*test was used. *P* values are shown as the exact values. Source data

We next examined antigen-specific responses. One month after injection of AAV-OVA (or hen-egg lysozyme (HEL)-OVA), we were able to detect OVA antigen in the brain homogenate and CSF, but not in the serum (Extended Data Fig. 6a,b). We adoptively transferred naive CD4^+^ T cells from OT-II mice and B cells from MD4 mice after 1 month of AAV injection, and analysed their activation in various organs 1 week later (Fig. 4a). Transferred cells were predominantly found in different tissues, with minimal presence in the circulation (Extended Data Fig. 6c).

HEL-OVA expression in neurons resulted in minor changes in overall immune cell numbers in the skull BM (Extended Data Fig. 6d–f). Notably, skull OT-II T cells were activated, as indicated by CD44 expression with enhanced differentiation into T_FH_ cells. MD4 B cells also displayed more GC marker expression compared with the control group (Fig. 4b,c and Extended Data Fig. 6g,h). Similarly, neuronal HEL-OVA expression did not change the overall immune cell numbers in whole-brain tissues, while the number and activation marker expression of OT-II and MD4 cells were increased compared with the control group (Extended Data Fig. 6i,j). Transferred cells were not detected within the brain parenchyma (Extended Data Fig. 6k). Moreover, no significant differences in OT-II and MD4 cells were observed in the dura, dCLNs, spleen or sternum BM (Extended Data Fig. 6l–o). These findings suggest that neuron-derived antigens can specifically activate and differentiate T and B cells in the brain and skull BM under non-disease conditions.

**Extended Data Fig. 7:**
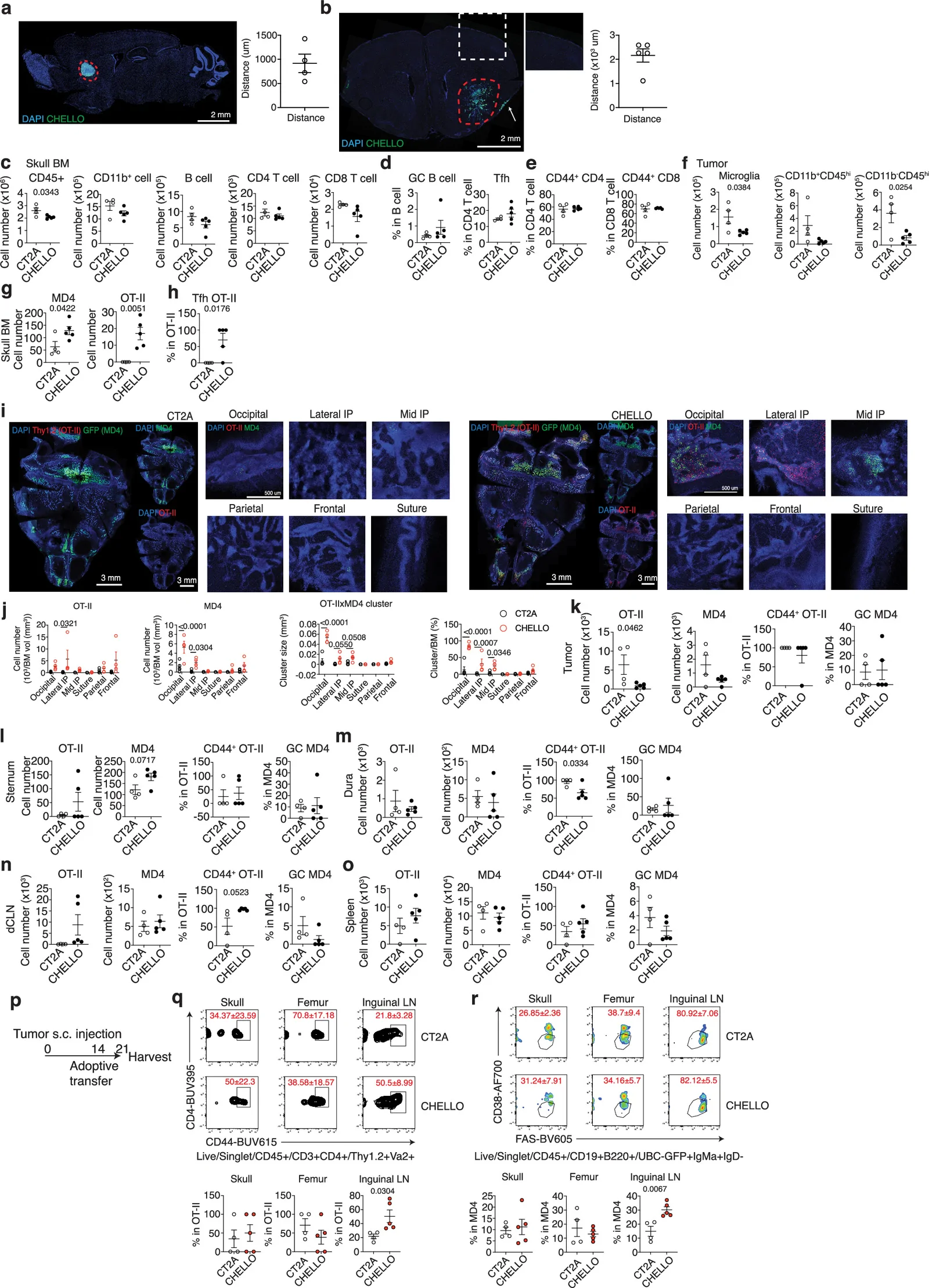
Systemic immune responses in tumour models. **a**. Sagittal section of tumour bearing brain 14 days post injection (left): tumour mass (red circle). Distance between tumours and dorsal leptomeninges (right). n = 4 of mice were used. **b**. Coronal brain sections: tumour mass (red circle), zoom (white box, middle), quantification of tumour-meninges distance (right). n = 5 mice were used. Leptomeningeal invasion (white arrow). **c-e**. Fourteen days post intracranial CT2A or CHELLO injection, naïve OT-II CD4 T cells and MD4 B cells were adoptively transferred; one week later, skull BMs analysed; immune cell numbers (**c**), frequencies of differentiated B and T cells (**d**), activated CD4 and CD8 T cells (**e**). n = 4 (CT2A) and n = 5 (CHELLO) mice were used. **f**. Flow cytometry of tumour-bearing brain immune cells. Number of microglia, CD11b^+^CD45^hi^, and CD11b^-^CD45^hi^ cells. n = 4 (CT2A) and n = 5 (CHELLO) mice were used. **g-h**. Fourteen days post intracranial CT2A and CHELLO injection, naïve OT-II CD4 T cells and MD4 B cells were adoptively transferred; one week later, skull BMs analysed; the number of MD4 B cells and OT-II CD4 T cells (**g**), and frequency of Tfh cells among OT-II CD4 T cells (**h**). n = 4 (CT2A) and n = 5 (CHELLO) mice were used. **i-j**. Two weeks post tumour injection, whole OT-II CD4 T cells and MD4 B cells were i.v. transferred; one week later, whole mount skulls were imaged by confocal microscopy (representative images of whole skull, and each area, **i**; and quantifications, **j**). n = 5 (CT2A) and n = 4 (CHELLO) mice were used. **k-o**. Analysis of MD4 B and OT-II CD4 T cells in tumour tissues (**k**), sternum BM (**l**), dura (**m**), dCLN (**n**), and spleen (**o**). n = 4 (CT2A) and n = 5 (CHELLO) of mice were used. **p-r**. Experimental scheme of peripheral tumour injection and adoptive transfer of naïve Thy1.2^+^ OT-II CD4 T cells and UBC-GFP MD4 B cells into Thy1.1 host mice (**p**). Frequency of CD44^+^ OT-II CD4 T cells (**q**) and GC MD4 B cells (**r**). n = 4 (CT2A) and n = 5 (CHELLO) of mice were used. Data are represented as mean ± s.e.m. Two-way ANOVA with Sidak’s multiple comparisons (**j**) and Two-tailed unpaired Student’s *t-*test (**c-h, k-r**) were used. *P* values are shown as the exact values. Source data

**Extended Data Fig. 8:**
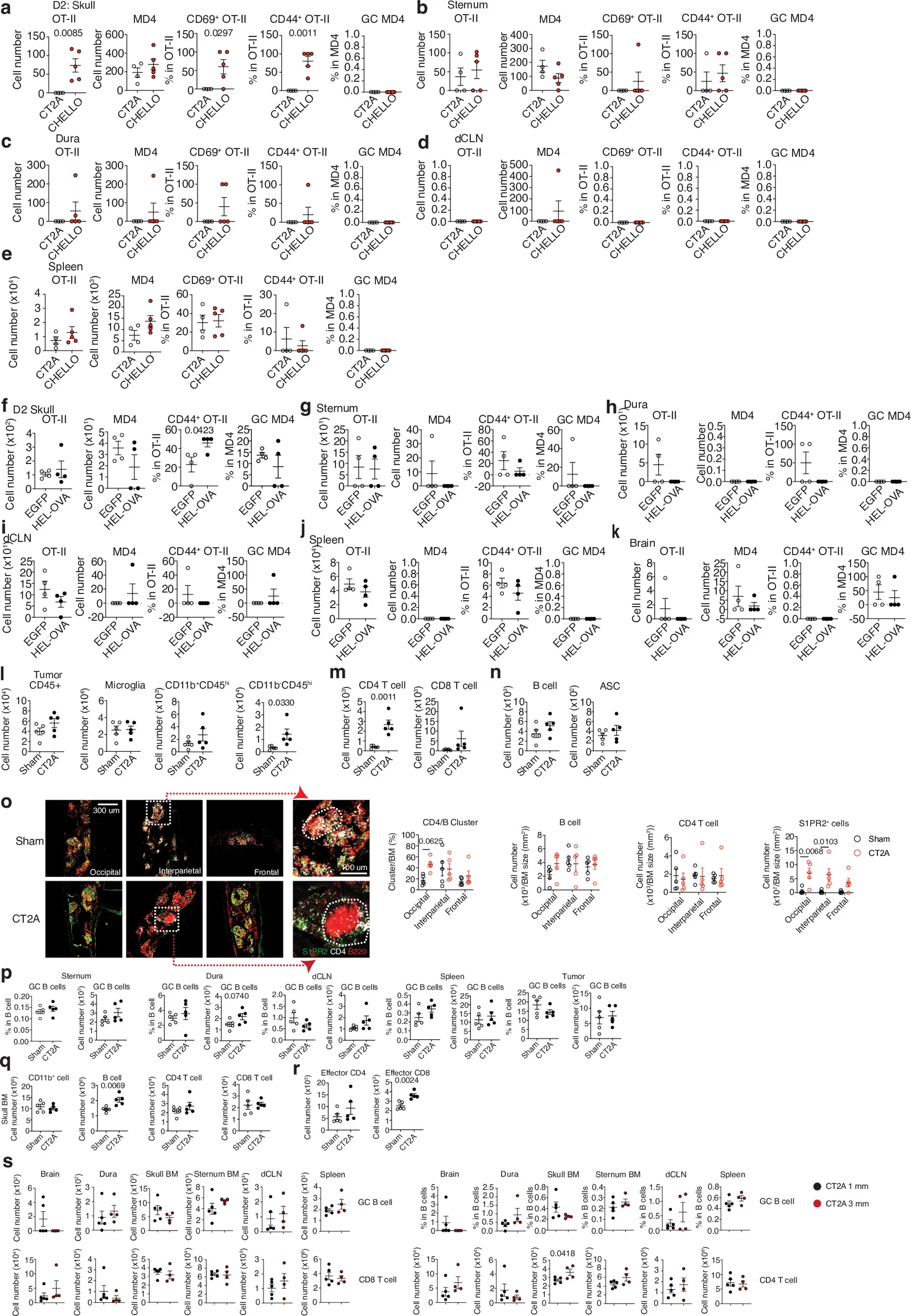
Early antigen-specific responses and tumour-induced skull responses. **a-e**. Fourteen days post CT2A or CHELLO injection, naïve OT-II CD4 and MD4 B cells were adoptively transferred; two days later, recruitment and activation were analysed in skull BM (**a**), sternum BM (**b**), dura (**c**), dCLN (**d**), and spleen (**e**). n = 4 (CT2A) and n = 5 (CHELLO) of mice were used. **f-k**. Four weeks post AAV-EGFP or AAV-HEL-OVA injection, naïve OT-II CD4 and MD4 B cells were adoptively transferred; two days later, recruitment and activation were analysed in skull BM (**f**), sternum BM (**g**), dura (**h**), dCLN (**i**), spleen (**j**), and brain (**k**). n = 4 mice per group were used. **l-n**. Fourteen days post sham or CT2A injection, brain tumour tissues were analysed; immune cell numbers (**l**), T cell subsets (**m**), B cells and ASCs (**n**). n = 5 mice per group. **o**. Confocal images of skull BM coronal sections two weeks post sham surgery or tumour injection; representative images (left), quantification (right). n = 5 of mice per group were used. **p**. Frequency and number of GC B cells in indicated tissues after sham surgery or CT2A injection. n = 5 mice per group. **q-r**. Number of indicated immune cells (**q**) and effector T cells (**r**) in the skull BM after sham surgery or CT2A injection. n = 5 of mice per group were used. **s**. After 2 weeks of 1 mm-depth CT2A or 3 mm-depth CT2A injection, tissues were harvested for flow cytometry analysis. The number and frequency of GC B cells, CD4 T cells, and CD8 T cells. n = 5 (1 mm) and n = 4 (3 mm) of mice were used. Data are represented as mean ± s.e.m. Two-way ANOVA with Sidak’s multiple comparisons (**o**) and Two-tailed unpaired Student’s *t-*test (**a-n, p-s**) was used. *P* values are shown as the exact values. Source data

We next examined antigen-specific T and B cell activation in the skull BM when antigens are derived from brain tumour cells. We engrafted either the syngeneic CT2A mouse glioma cell line or the CT2A line expressing HEL-OVA (CHELLO) into immunocompetent mice. First, we confirmed that tumour cells remained within the parenchyma without exiting along the injection route (notably, a subset of mice exhibited invasion into the proximal leptomeninges; Extended Data Fig. 7a,b). Second, CHELLO engraftment did not increase overall immune cell numbers in the skull BM or within the tumour mass when compared with the control CT2A group, suggesting that CHELLO engineering did not lead to notable immunogenicity (Extended Data Fig. 7c–f). However, after 1 week of OT-II and MD4 cell transfer, cell numbers and expression of activation markers of transferred cells in the skull BM were significantly increased (Fig. 4d–f and Extended Data Fig. 7g,h). Transferred cells were preferentially localized to the posterior skull BM (Extended Data Fig. 7i,j). CHELLO engraftment induced only minor changes in OT-II and MD4 cells in the tumour region and other tissues (Extended Data Fig. 7k–o). On the other hand, peripheral (into flank) CT2A injection elicited responses in the draining inguinal LNs but not the skull BM (Extended Data Fig. 7p–r). Thus, CNS antigen-specific anti-tumour T and B cell responses can occur locally in the skull BM. Moreover, when examined as early as day 2 after transfer, antigen-specific OT-II responses were detected only in the skull BM, whereas GC responses required a longer time to develop, suggesting that the skull BM is among the first brain barriers to respond to brain-derived antigens (Extended Data Fig. 8a–k).

Finally, we tested whether glioma grafts induce endogenous GC responses in the skull BM by comparing sham and CT2A-injected groups. CT2A injection increased the number of GC-like B cells in the skull BM, but it did not affect those in the sternum BM, dCLNs, spleen and brain, while the dura exhibited a modest increase in cell number (Fig. 4g and Extended Data Fig. 8l–p). Moreover, CT2A engraftment increased the number of B cells and effector CD8^+^ T cells in the skull BM (Extended Data Fig. 8q,r). Shallow tumour implantation, which enhances meningeal invasion, drove similar T and B cell responses in the skull BM in comparison to striatal tumours (Extended Data Fig. 8s).

**Extended Data Fig. 9:**
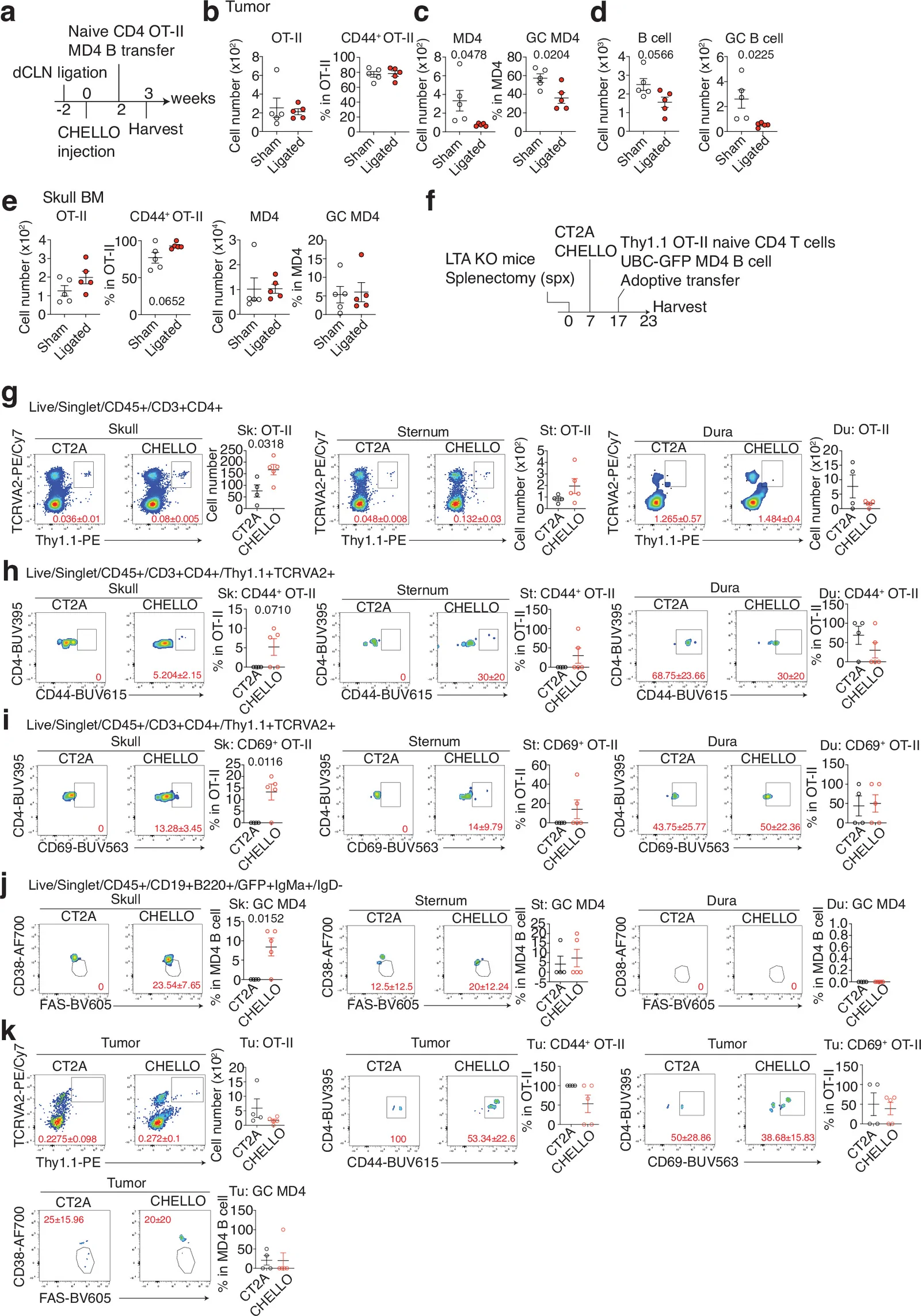
Tumour antigen-specific responses in LN- and spleen-deficient mice. **a-e**. Two weeks post sham or dCLN ligation, CHELLO cells were injected. Two weeks later, naïve OT-II::Thy1.1 CD4 T cells and UBC-GFP::MD4 B cells were adoptively transferred. One week later, tissues analysed (**a**): OT-II (**b**), MD4 (**c**), and polyclonal B cells in tumour tissues (**d**), OT-II and MD4 in skull BM (**e**). n = 5 of mice per group were used. **f-k**. Experimental scheme (**f**). Numbers of OT-II CD4 T cells (**g**), CD44 expression (**h**), CD69 expression (**i**), GC MD4 B cells (**j**) in indicated tissues, and these cells in tumour tissues (**k**). n = 4 (CT2A) and n = 5 (CHELLO) of mice were used. Data are represented as mean ± s.e.m. Two-tailed unpaired Student’s *t-*test was used. *P* values are shown as the exact values. Source data

To determine whether skull lymphoid responses occur independently of SLO responses, we first performed dCLN ligation surgery in CHELLO mice (Extended Data Fig. 9a). While OT-II responses in the tumour grafts were not affected by dCLN ligation, the cell number and activation of tumour-infiltrating MD4 B cells and total polyclonal B cells were reduced, suggesting that dCLNs support intratumoural B cell responses (Extended Data Fig. 9b–d). However, the cell number and activation of OT-II CD4^+^ T cells and MD4 B cells in the skull BM were not reduced by dCLN ligation (Extended Data Fig. 9e). Moreover, antigen-specific T and B cell responses were still detected in the skull BM of splenectomized lymphotoxin-α (LTA)-deficient mice (Extended Data Fig. 9f–k). These data suggest that immune responses can occur within the skull lymphoid structures, including those against local antigens, in the absence of SLOs.

**Extended Data Fig. 10:**
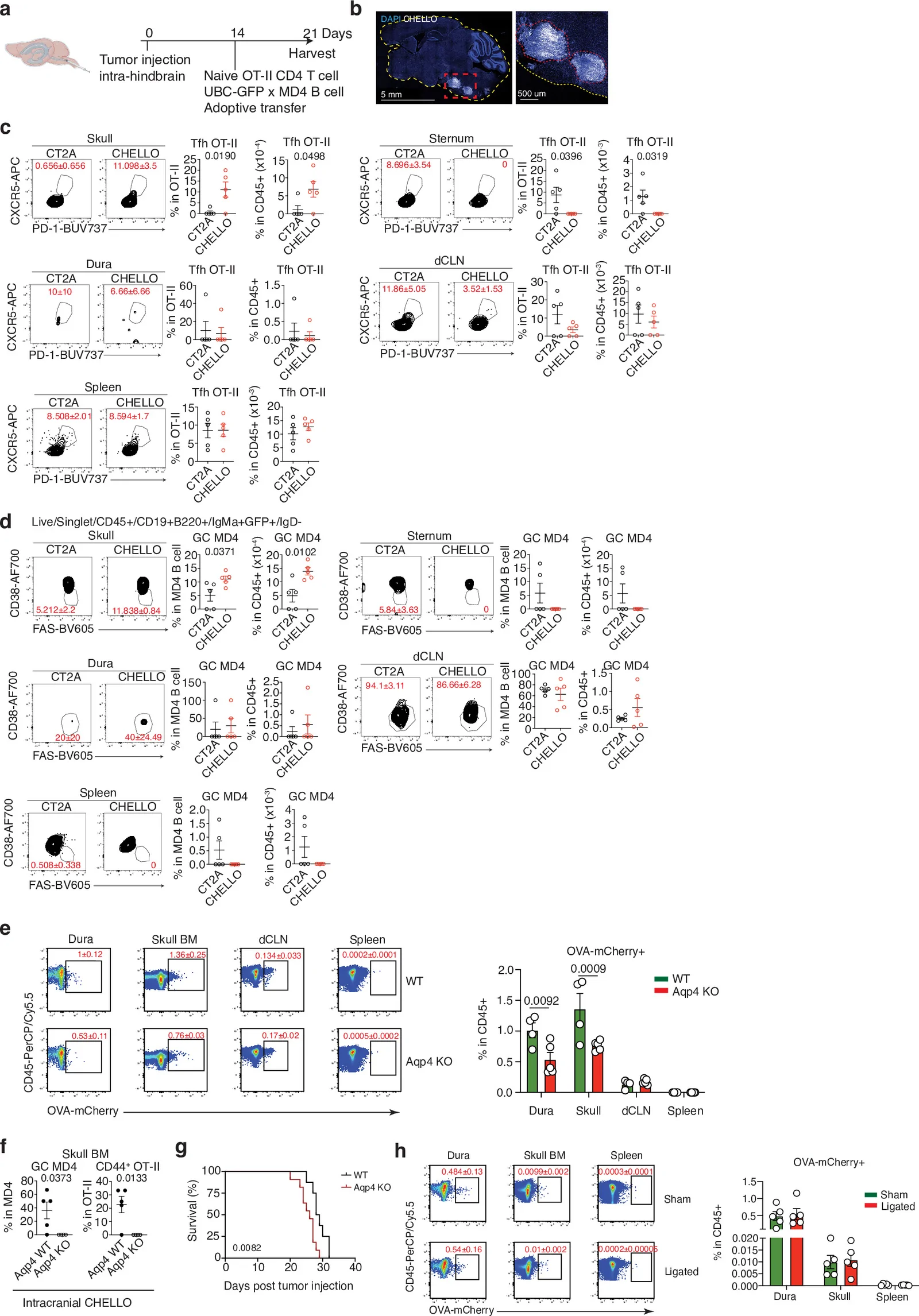
Tumour antigen-specific responses are not mediated by skull damage. **a**. Experimental scheme for intra-hindbrain tumour injection and adoptive transfer. **b**. Sagittal tumour-bearing brain section with zoomed tumour mass (red box, right). **c-d**. Frequency of Tfh cells among transferred OT-II cells (**c**) and GC cells among MD4 B cells (**d**). n = 5 of mice per group were used. **e**. After 4 weeks of intracranial AAV-hSyn-OVA-mCherry-P2A-EGFP injection into WT or Aqp4 KO mice, each tissue was harvested for flow cytometry analysis. n = 4 (WT) and n = 5 (KO) mice were used. **f**. Frequency of GC MD4 cells and CD44^+^ OT-II cells of skull BM after 1 week of transfer from CHELLO-injected (post 2 weeks) Aqp4 WT (n = 5) or KO (n = 4) mice. **g**. Survival of WT and Aqp4 KO mice after CT2A tumour implantation. n = 8 (WT) and n = 11 (KO) mice were used. **h**. After 1 week of intracranial AAV-hSyn-OVA-mCherry-P2A-EGFP injection, mice received sham or dCLN ligation surgery. After 2 weeks of surgery, tissues were harvested for flow cytometry analysis. n = 5 mice per group. Data are represented as mean ± s.e.m. Two-tailed unpaired Student’s *t-*test (**c-d, f**) and two-way ANOVA with Sidak’s multiple comparison (**e, h**) were used. For survival analysis, log-rank test was used (**g**). *P* values are shown as the exact values. The illustration in **a** was created using BioRender; Kipnis, J. https://BioRender.com/f3anzmq (2026). Source data

To rule out the possibility that craniotomy injury contributes to our observations, we performed intrahindbrain tumour injection through the cisterna magna without craniotomy, which reproduced the previous results of skull-specific OT-II/MD4 responses (Extended Data Fig. 10a–d).

To address the role of antigen clearance from the brain in driving immune response, we used *Aqp4*-deficient mice, which are impaired in CSF perfusion of the brain (glymphatic system)^30^. *Aqp4*-deficient mice showed reduced antigen uptake in the dura and skull BM, impaired antigen-specific responses in the skull BM and decreased survival in the CT2A brain tumour model (Extended Data Fig. 10e–g), whereas dCLN ligation did not affect antigen uptake in these tissues (Extended Data Fig. 10h). These data suggest that active, glymphatic-driven antigen efflux from the brain is required for skull lymphoid responses.

In summary, immune cells in the skull BM can be activated and expand GC-like structures in response to brain-derived antigens.

## Anti-tumour immunity

**Fig. 5:**
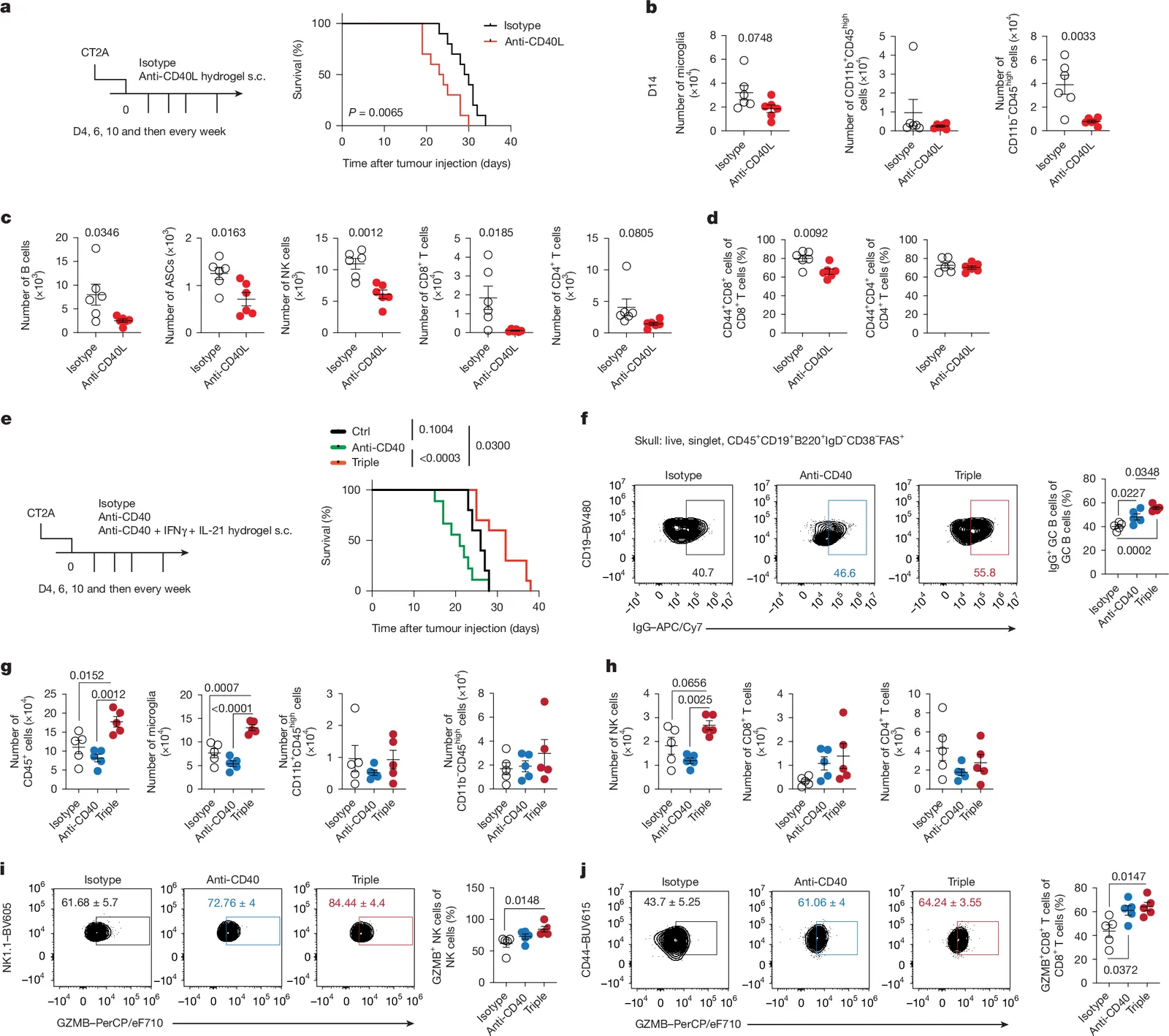
Skull lymphoid structure contributes to anti-tumour immune responses. **a**, Experimental schematic (left) and Kaplan–Meier survival plot (right) for s.c. injection of anti-CD40L and intracranial tumour injection. *n* = 10 mice per group. **b**,**c**, Flow cytometry analysis (day 14) of the numbers of each immune cell type. *n* = 6 mice per group. **d**, The percentage of CD44^+^ activated cells in CD8^+^ and CD4^+^ T cells. *n* = 6 mice per group. **e**, Experimental schematic (left) and Kaplan–Meier survival plot (right) for triple therapy (anti-CD40 + IFNγ + IL-21 injection). *n* = 5 (ctrl), *n* = 9 (anti-CD40) and *n* = 10 (triple therapy) mice. **f**–**h**, Analysis of immune responses at 14 days after tumour injection with the indicated therapy. **f**, The percentage of IgG^+^ cells in GC B cells of skull BM. **g**, The number of total CD45^+^ immune cells, microglia (CD45^low^CD11b^+^), CD11b^+^CD45^high^ myeloid tumour-infiltrating leukocytes and CD11b^−^CD45^high^ lymphocytes. **h**, The number of NK cells, CD8^+^ T cells and CD4^+^ T cells in tumour tissues. *n* = 5 mice per group. **i**,**j**, Intracellular cytokine analysis at day 14 after restimulation with PMA and ionomycin for 4 h, showing the percentage of GZMB-expressing cells in NK cells (**i**) and CD8^+^ T cells (**j**). *n* = 5 mice per group. Survival data were analysed using the log-rank test (**a** and** e**). Holm–Sidak post hoc analysis was applied (**e**) for multiple-comparison correction. Two-tailed unpaired Student’s *t*-tests were used for **b**–**d**, and one-way ANOVA with Tukey’s multiple-comparison test was used for **f**–**j**. Data are mean ± s.e.m. Exact *P* values are shown. Source data

**Extended Data Fig. 11:**
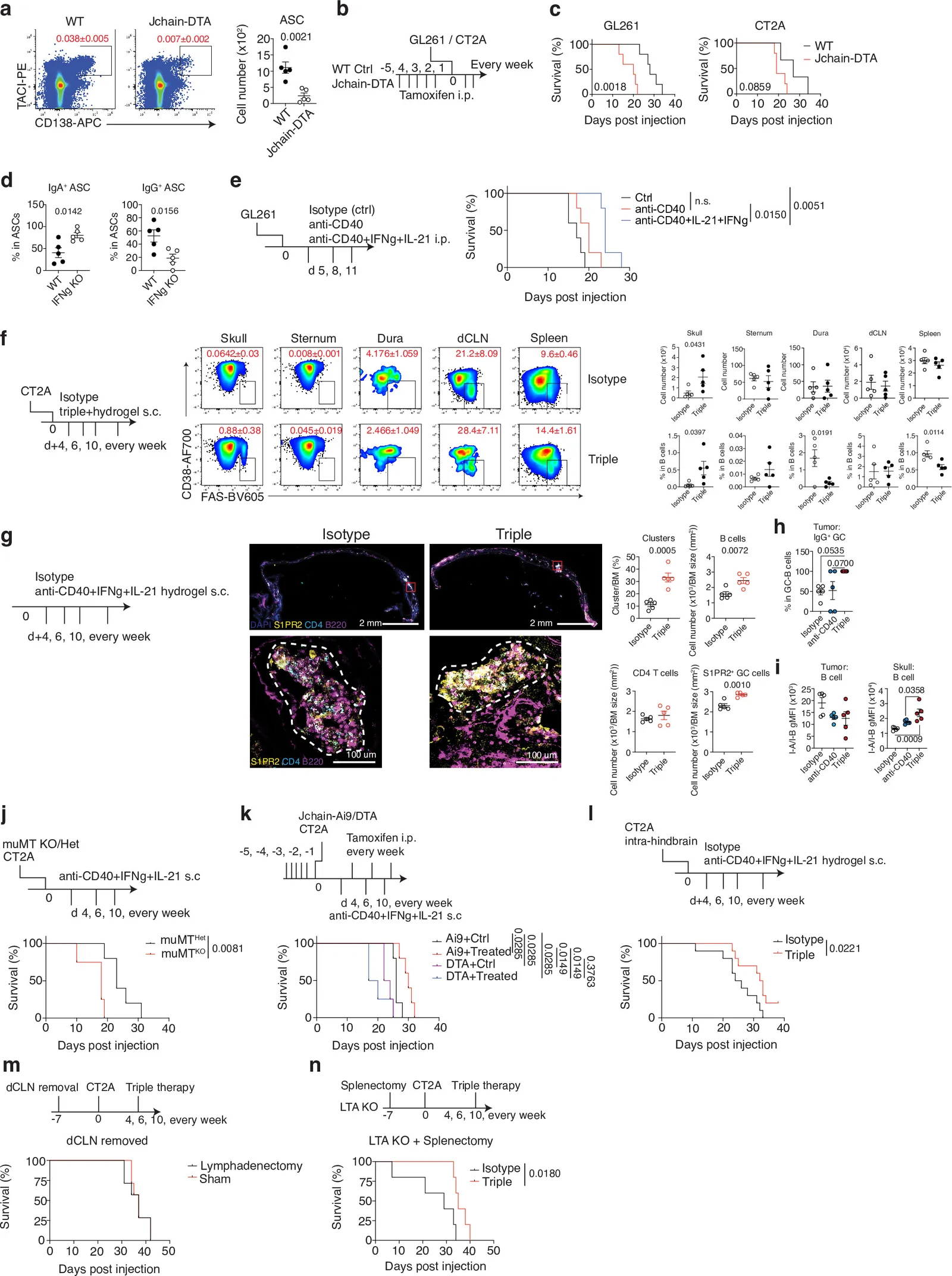
Humoral immunity is required for antitumor immune responses. **a**. ASC depletion confirmed by flow cytometry after intraperitoneal tamoxifen injection in WT and Jchain-CreERT2::ROSA-DTA mice (Jchain-DTA; FACS plots, left; ASC numbers, right). n = 5 of mice were used per group. **b-c**. Experimental scheme (**b**) and Kaplan-Meier survival curves of GL261- or CT2A-bearing WT versus Jchain-DTA mice (**c**). n = 5 (GL261, WT), n = 5 (GL261, Jchain-DTA), n = 3 (CT2A, WT), and n = 5 (CT2A, Jchain-DTA) of mice were used. **d**. Frequency of IgA^+^ and IgG^+^ ASCs in skull BM from WT and IFNg KO mice. n = 5 of mice were used per group. **e**. Experimental scheme (left) and survival of systemic therapy-treated mice. (right). n = 5 of mice were used per group. **f**. The number and frequency of GC B cells in tissues following CT2A injection with isotype control (n = 5) or triple therapy (n = 5). **g**. Representative posterior skull BM sections with zooms (left) and quantification (right) after treatment. White dashed circle indicates BM. n = 5 mice per group. **h-i**. Flow cytometry after 14 days of CT2A injection with local isotype control, anti-CD40 or triple therapy treatment: IgG^+^ GC B cells in tumour tissues (**h**) and MHCII gMFI in tumour versus skull B cells (**i**). n = 5 mice per group. **j**. Survival of CT2A-bearing muMT heterozygous or homozygous mice after therapy. n = 5 (muMT-het), n = 4 (muMT-KO) of mice were used. **k**. Survival of CT2A-bearing Jchain-Ai9 or Jchain-DTA mice treated with therapy or control. n = 5 (WT+Ctrl and WT+Triple) and n = 4 (DTA+Ctrl and DTA+Triple) of mice were used. **l**. Survival after intra-hindbrain CT2A injection with therapies. n = 10 of mice per group were used. **m**. After 1 week of dCLN removal (lymphadenectomy) or sham surgery, CT2A tumours were implanted. Mice were treated with local triple therapy at indicated days. n = 7 mice per group were used. **n**. Survival of local isotype control- or triple therapy-treated LTA KO mice with splenectomy surgery. n = 5 mice per group were used. Two-tailed unpaired Student’s *t*-test (**a, d, f, g**) were used. One-way ANOVA with Tukey’s multiple test was used (**h, i**). Survival data (**c, e, j-n**) were analysed with log-rank test. Holm-Sidak post-hoc analysis was used for multiple comparison (**e, k**). Data are represented as mean ± s.e.m. *P* values are shown as the exact values. Source data

Finally, we investigated whether skull lymphoid structures have a functional role in models of brain tumours. Although B cells are known to have mixed roles in brain tumours^31,32^, we confirmed that antibody responses are essential for optimal anti-tumour responses by depleting ASCs (Extended Data Fig. 11a–c). Subcutaneous delivery of hydrogel containing anti-CD40L antibodies disrupted skull lymphoid responses (Supplementary Fig. 3d–j). This partial blockade of skull lymphoid structures significantly reduced overall survival in CT2A-bearing mice (Fig. 5a), accompanied by decreased lymphocyte infiltration (Fig. 5b,c) and attenuated intratumoural CD8^+^ T cell activation (Fig. 5d).

To examine the therapeutic potential of skull BM lymphoid structures, we enhanced this niche using a CD40 agonist antibody. As CD40 agonism alone is known to induce regulatory B cells^33^, we added IL-21 and IFNγ to promote GC and IgG responses, respectively (Supplementary Fig. 2d–h and Extended Data Fig. 11d). Systemic administration of the CD40 agonist alone led to a slight, non-significant increase in survival of CT2A glioma-bearing mice; however, administering all three agents together significantly extended their survival (Extended Data Fig. 11e). Local administration of a CD40 agonist mixed with cytokines and hydrogel under the scalp enhanced skull lymphoid responses but not in other tissues (Extended Data Fig. 11f,g), and significantly prolonged survival (Fig. 5e). However, local treatment with anti-CD40 agonist alone resulted in earlier mortality, possibly due to the induction of regulatory B cells. This local triple therapy also successfully induced B cell activation and IgG^+^ GC responses in the skull BM, while the change in intratumoural B cell activity was minor (Fig. 5f and Extended Data Fig. 11h,i), suggesting that this local treatment primarily targets skull BM B cells. Moreover, it was ineffective in B-cell-deficient muMT mice and ASC-deficient Jchain-DTA mice, indicating that its efficacy relies on B cells and antibody responses of ASCs (Extended Data Fig. 11j,k). Local triple therapy also exerted anti-tumour effects in mice with craniotomy-free intrahindbrain tumour injections, indicating that these results were not driven by surgery-induced damage artefacts (Extended Data Fig. 11l). Moreover, the efficacy of treatment was not affected by dCLN removal (Extended Data Fig. 11m), and this local treatment prolonged survival in splenectomized LTA-deficient mice, suggesting that skull BM-directed local therapy can retain therapeutic efficacy even when classical SLO function is compromised (Extended Data Fig. 11n). However, control splenectomized LTA-deficient mice exhibited shorter survival compared with wild-type (WT) mice (Fig. 5e and Extended Data Fig. 11n), and antigen-specific responses were generally weaker in magnitude than those of WT mice (Fig. 4d–f and Extended Data Fig. 9f–k).

Taken together, these results suggest that skull BM contains early-responding functional lymphoid structures that participate in anti-tumour immunity independently of peripheral SLOs, while the peripheral immune compartments contribute at later stage to amplify and sustain these responses.

**Extended Data Fig. 12:**
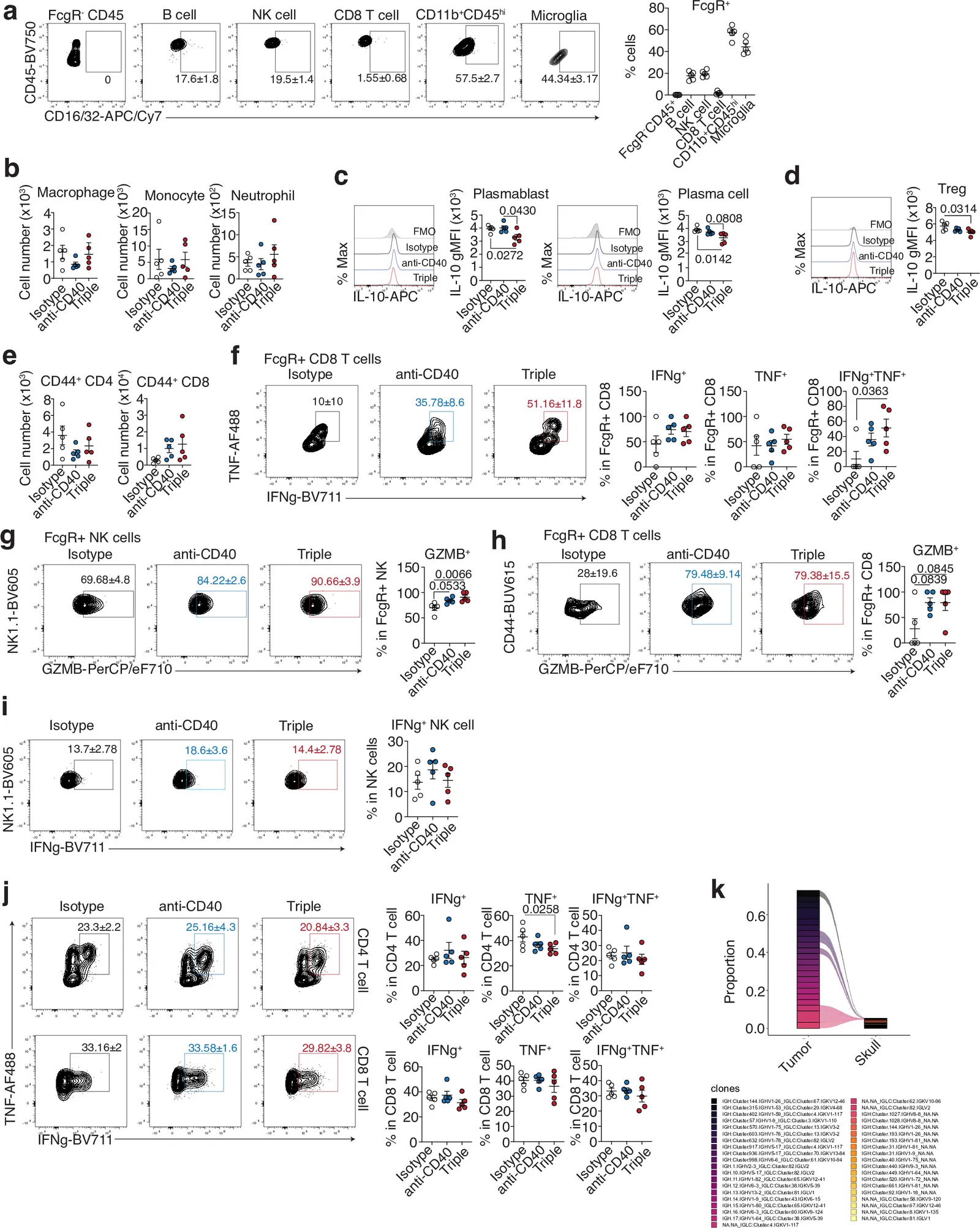
Local triple therapy-induced antitumor immunity. **a**. Frequency of FcgR (CD16/32)-expressing subsets among CT2A tumour-infiltrating immune cells (14 days post injection). n = 5 mice were used. **b**. Flow cytometry analysis after 14 days of CT2A injection with therapies for myeloid subsets in tumour tissues. n = 5 mice per group. **c-d**. Tumour-derived cells stimulated ex vivo with PMA/ionomycin: gMFI of IL-10 in plasmablast/plasma cells (**c**) and Tregs (**d**). n = 5 mice per group. **e**. Flow cytometry analysis after 14 days of CT2A injection with therapies for activated T cells in tumour tissue. n = 5 mice per group. **f-j**. Tumour-derived cells stimulated ex vivo with PMA/ionomycin: IFNg^+^/TNF^+^ FcgR^+^ CD8 T cells (**f**); GZMB^+^ FcgR^+^ NK cells (**g**) and CD8 T cells (**h**); IFNg^+^ cells in NK cells (**i**); IFNg^+^/TNF^+^ CD4 and CD8 T cells (**j**). n = 5 of mice were used per group. One-way ANOVA with Tukey’s multiple test was used. Data are represented as mean ± s.e.m. *P* values are shown as the exact values. **k**. Alluvial plot showing shared BCRs between tumour tissues and skull BM. Source data

In the tumour microenvironment, diverse immune cells, including myeloid cells, B cells, natural killer (NK) cells and CD8^+^ T cells, expressed the Fcγ receptor (FcγR; CD16/32), potentially mediating IgG-driven immune responses (Extended Data Fig. 12a). The triple-therapy group had an increased number of overall intratumoural CD45^+^ immune cells and microglia, while the CD45^high^ myeloid composition remained largely unchanged (Fig. 5g and Extended Data Fig. 12b).

Triple therapy reduced IL-10 expression of several immune cells such as PBs, PCs and T_reg_ cells (Extended Data Fig. 12c,d), and markedly enhanced cytotoxicity of NK cells and CD8^+^ T cells, as evidenced by elevated GZMB expression, while changes in the total cell number were minor (Fig. 5h-j and Extended Data Fig. 12e). Moreover, increased polyfunctionality by triple therapy treatment was specifically observed in FcγR^+^CD8^+^ T cells (Extended Data Fig. 12f). FcγR^+^ NK and CD8^+^ T cells also exhibited enhanced GZMB expression (Extended Data Fig. 12g,h), while overall cytokine production of total NK and T cells was comparable across groups (Extended Data Fig. 12i,j), potentially suggesting FcγR-mediated activation. Given the shared BCR repertoire between tumour-infiltrating and skull BM PNA^+^ B cells, these findings suggest that intratumoural immunity is, at least in part, shaped by skull-derived B cell responses, potentially through IgG–FcγR interactions (Extended Data Fig. 12k).

Overall, these data indicate that skull-targeting CD40 agonism combined with administration of the IL-21 and IFNγ supports IgG responses in the skull BM and subsequently enhances intratumoural microglial, NK and CD8^+^ T cell anti-tumour responses while attenuating suppressive immune cells.

## Discussion

Here we found functional lymphoid structures within the skull BM, encompassing T_FH_ cells, FDCs and GC-like structures. These structures respond to CNS-derived antigens and mount antigen-specific adaptive immune responses in disease-free and brain tumour model context, thereby partaking in CNS immune surveillance.

The skull BM is a primary lymphoid tissue that supplies immune cells, including myeloid cells and immature B cells, to the brain and its border regions^5,6^. BM is also known to contain mature immune cells, including memory and regulatory T cells^9,13,19^. Although the presence of these cells suggests that BM can support adaptive immune responses against circulating antigens^14,16,18^—particularly during inflammation^8,11,15^, or when peripheral SLOs are nonfunctional^10^—such responses are generally limited under healthy conditions. Here we demonstrate that lymphoid structures, including GCs, are localized within the skull BM, not observed to the same extent in other BM sites. T_FH_ cells promote constitutive B cell activation through cytokines and co-stimulatory molecules, enabling a sustained immune activity. This activity may be facilitated by continuous exposure to CNS-derived antigens delivered via the CSF through osseous channels^1–4^.

Our OVA–mCherry-tracing experiments showed that brain-derived antigen can reach the skull BM, where it supports local antigen presentation within lymphoid structures. OVA distribution along perivascular spaces and brain borders, together with AQP4 dependency, supports drainage through physiological glymphatic pathways. Selective OVA detection in the skull BM, but not in the sternum BM or spleen, suggests likely preferential use of skull channels^1–4^ rather than systemic dissemination, although the latter cannot be fully excluded. In the dCLNs, OVA was detected using whole-mount imaging but was not robustly detected within immune cells by flow cytometry. This discrepancy may reflect limited internalization, fluorophore loss or degradation during longer-distance drainage. The limited OVA accumulation in the dCLNs compared with previous intracisternal tracer studies may reflect differences between tracer delivery into the CSF and physiological antigen release from the brain parenchyma, as also discussed previously^34^. Indeed, ref. ^34^ similarly observed accumulation of neuron-derived proteins in the dCLNs by imaging with limited intracellular detection, and found that their drainage patterns differed from those of injected tracers. Their study proposed a nearest-exit principle whereby CNS-derived proteins preferentially drain toward proximal border compartments. Although striatal antigens preferentially used basal and nasal routes, this does not preclude access to the posterior skull BM, where OVA was directly detected in our study. Moreover, ref. ^34^ found slower tracer turnover in the skull BM than in other regions, suggesting prolonged antigen retention. Together with pre-existing lymphoid structures enriched in the occipital skull BM, these findings suggest that, in addition to antigen drainage, the local immune environment may further facilitate antigen-specific immune responses.

While our data indicate that skull lymphoid structures are among the earliest responders to brain-derived antigens and contribute substantially to CNS immunosurveillance and anti-tumour immunity, the contribution of other border tissues and peripheral organs cannot be excluded. SLO-deficient mice still permitted antigen-specific responses within the skull BM and retained therapeutic responses to skull-targeting triple therapy. However, these responses were reduced in magnitude, and tumour-bearing SLO-deficient mice exhibited shorter survival compared with WT mice. These data suggest that skull BM provides rapid immune responses, potentially owing to its close anatomical proximity to the CNS, while peripheral immune compartments may contribute to amplification and propagation of the responses. Further studies are needed to define the complex interactions among CNS border tissues and peripheral immune compartments, as well as their relative contributions and functional hierarchy within the neuroimmune axis.

Recent work identified effector memory CD8^+^ T cells in the skull BM of patients with glioblastoma^8^. These cells possess cytotoxic functionality and share TCRs with tumour-infiltrating T cells, suggesting that the skull BM may participate in anti-tumour immune responses in humans. The presence of T_FH_-like cells in human skull BM further supports the potential relevance of these findings to human biology; however, additional studies will be required to determine how these mechanisms translate to human patients. In particular, the magnitude and organization of skull lymphoid responses may vary depending on disease contexts, including tumour location, meningeal involvement and patterns of CNS antigen drainage. As shown in our data, a subset of mice exhibited leptomeningeal invasion as tumours progressed. The extent of such involvement may vary depending on tumour type, location and growth kinetics. Although our comparison of shallow tumours, which increase the likelihood of meningeal involvement, and striatal tumours did not reveal differences in overall T and B cell responses across tissues, tumour invasion into the meninges may directly alter border immune microenvironments and antigen drainage pathways, potentially leading to immune responses distinct from those observed in tumours confined to the parenchyma. Defining how meningeal involvement influences antigen delivery and skull-associated responses will therefore be important for understanding the broader applicability of our findings. Future studies across broader pathological settings and human specimens will be also important to define the generalizability and clinical relevance of these responses.

In conclusion, this study identifies functional lymphoid structures in the skull BM that support CNS antigen-specific immune responses under both homeostatic and pathological conditions. Our findings suggest that the skull BM represents an adaptive immune niche at the CNS borders and may provide insights into neuroimmune interactions and inform future therapeutic strategies for brain tumours and other neurological diseases.

## Methods

### Animals

SPF WT C57BL/6J (JAX, 000664) mice were obtained from The Jackson Laboratory. The following strains were also purchased from The Jackson Laboratory: *Il21-VFP* (*B6.Cg-Il21*^*tm1.1Hm*^*/DcrJ*, JAX, 030295)^35^, *Il21r*-knockout (*B6.129-Il21r*^*tm1Kop*^*f/J*, JAX, 019115)^36^, OT-II (*B6.Cg-Tg(TcraTcrb)425Cbn/J*, JAX, 004194)^37^, MD4 (*C57BL/6-Tg(IghelMD4)4Ccg/J*, JAX:002595)^38^, *Jchain-creERT2* (*B6(129)-Jchain*^*em1(icre/ERT2)Deep*^*/J*, JAX, 035764)^39^, ROSA-DTA (*B6.129P2-Gt(ROSA)26Sor*^*tm1(DTA)Lky*^*/J*, JAX, 009669)^40^, Ai9 (*B6.Cg-Gt(ROSA)*^*26Sortm9(CAG-tdTomato)Hze*^*/J*, JAX, 007909)^41^, Thy1.1 (*B6.PL-Thy1a/CyJ*, JAX, 000406), AID-cre (*B6.129P2-Aicda*^*tm1(cre)Mnz*^*/J*, JAX, 007770)^42^, Ai14 (*B6.Cg-Gt(ROSA)26Sor*^*tm14(CAG-tdTomato)Hze*^*/J*, JAX, 007914)^41^, UBC-GFP (*C57BL/6-Tg(UBC-GFP)30Scha/J*, JAX, 004353)^43,44^, *Ifng* KO (*B6.129S7-Ifng*^*tm1Ts*^*/J*, JAX, 002287)^45^, *Lta* KO (*B6.129S2-Lta*^*tm1Dch*^*/J*, JAX, 002258)^46^, *Aqp4* KO (*B6(Cg)-Aqp4<tm1.1Tsna>*, RIKEN, RBRC10053)^47^ and muMT (*B6.129S2-Ighm*^*tm1Cgn*^*/J*, JAX, 002288)^48^ mice. All animals were housed in a temperature-controlled (22 °C) and humidity-controlled (33–39%) environment under a 12 h–12 h light–dark cycle and had ad libitum access to food and water.

Gnotobiotic mice were housed in plastic flexible film isolators in the gnotobiotic facility, also under a 12 h–12 h light–dark cycle. These mice were weaned onto autoclaved food, with ad libitum access to food and water. GF mice were fed γ-irradiated AAD (Teklad custom diet, TD.130916) for 4 weeks to establish AAD-fed GF cohort. Unless otherwise specified, 8-week-old male mice were used for experiments. All procedures were approved by the Institutional Animal Care and Use Committee of Washington University in St Louis (23-0145).

### Cell lines and AAVs

The mouse brain cancer cell line GL261 was provided by G. Dunn^49^. The mouse glioma cell line CT2A was purchased from Sigma-Aldrich (SCC194). To generate HEL-OVA-expressing CT2A cell lines, full-length OVA DNA was obtained from Addgene (64599). The DNA encoding HEL-linker-OVA-IRES-eGFP was inserted into pLX208 vectors, and the cell lines were transduced with lentiviruses. Cells were selected with 100 µg ml^−1^ hygromycin (Invitrogen) and maintained with 50 µg ml^−1^ hygromycin supplementation.

Cell lines were passaged using trypsin/EDTA (Gibco, 25300) and maintained in Dulbecco’s modified Eagle’s medium (DMEM, Corning, 10-013-CM) supplemented with 10% FBS (Gibco, 26140) and 1% penicillin–streptomycin (Gibco, 15140). All cell lines were confirmed to be free of mycoplasma contamination. Cell lines were authenticated by short-tandem-repeat analysis.

pENN-AAV-hSyn-cre-WPRE-hGH (Addgene, 105553-AAV9), rAAV2/2-hSyn-DIO-eGFP-WPRE-hGH, rAAV2/2-hSyn-DIO-OVA-Flag-2A-eGFP-PA, rAAV2/9-hSyn-DIO-OVA-HEL-2A-eGFP-PA (Biohippo), AAV2-hSyn-OVA-P2A-eGFP-WPRE3-SV40polyA, and AAV2-hSyn-OVA-mCherry-P2A-eGFP-WPRE3-SV40polyA (AAVnerGene) were used.

### Tumour and AAV injections

For intracranial and intrahindbrain injection, mice were anaesthetized and secured in a stereotaxic instrument (Kopf) using isoflurane (5% for induction, 1–2% for maintenance) or an intraperitoneal (i.p.) injection of a ketamine (100 mg per kg) and xylazine (10 mg per kg) cocktail. Ophthalmic solution was applied to the eyes to prevent dehydration.

For intracranial AAV injection, after a small craniotomy, 0.5 μl of 1 × 10^12^ viral genomes per ml of AAVs diluted in Dulbecco’s phosphate-buffered saline (DPBS; Gibco, 14190) was injected into the striatum (coordinates: anteroposterior (AP), 1.5 mm; mediolateral (ML), 1.5 mm; dorsoventral (DV), −2.5 mm) using a Nanoliter 2020 injector (World Precision Instruments) with a fine glass capillary at a rate of 100 nl min^−1^.

For the intracranial tumour injection model, 1 × 10^5^ cancer cells in 2 μl of DPBS were injected into the brain (coordinates: AP, 2 mm; ML, 2 mm; DV, −3 mm) using a 26 G syringe (Hamilton, 80300) and an injector (KD Scientific)^50,51^. After intracranial injection, the burr hole was sealed with bone wax (Surgical Specialities Corporation, 901). For intrahindbrain injection, the posterior scalp and neck of anaesthetized mice were shaved. The head was secured in a stereotaxic frame with the neck tilted. A midline incision was made, and the posterior nuchal musculature was divided to expose the occipital bone and the dura overlying the cisterna magna. In total, 1 × 10^5^ cancer cells suspended in 2 μl of DPBS were injected into the hindbrain with a 26 G syringe through the cisterna magna. For peripheral tumour injection, anaesthetized mice were shaved, and 5 × 10^5^ cancer cells in 100 μl of DPBS were injected s.c. into the flank.

### Injections

For tamoxifen-induced Cre recombination, recipient mice were injected i.p. with 0.1 mg per g of tamoxifen (Sigma-Aldrich, T5648) dissolved in corn oil per day for five consecutive days. For T cell depletion, mice were treated with 200 μg of anti-CD4 (GK1.5, BioXcell, BE0003-1), anti-CD8a (2.43, BioXcell, BE0061) or rat IgG2b isotype control antibody (LTF-2, BioXcell, BE0090). For GC enhancement, 100 μg (i.p.) or 20 μg (s.c.) of anti-CD40 antibody (FGK4.5, BioXcell, BE0016) or rat IgG2a isotype control antibody (2A3, BioXcell, BE0089) was administered, with or without 10 μg (i.p.) or 2 μg (s.c.) of recombinant IFNγ (BioLegend, 575308) and 1 μg (i.p.) or 0.2 μg (s.c.) of recombinant IL-21 (BioLegend, 574506), either i.p. or transcranially. For GC blockade, 200 μg (i.p.) or 20 μg (s.c.) of anti-CD40L antibody (MR-1, BioXcell, BE0017-1) or polyclonal Armenian hamster IgG control antibody (BioXcell, BE0091) was injected i.p. or transcranially. For transcranial injections, materials were mixed with 10 mg ml^−1^ of carboxymethylcellulose hydrogel (Sigma-Aldrich) in a total volume of 300 μl and injected s.c. under the scalp. For peripheral injections, anti-CD40L antibody was mixed with hydrogel and injected s.c. into the flank.

For intracranial OVA injections, 0.5 μl of 1 mg ml^−1^ OVA-647 (Invitrogen) in DPBS was injected into the striatum in 1 min (coordinates: AP, 1.5 mm; ML, 1.5 mm; DV, −2.5 mm). For intracisternal OVA injections, 5 μl of 1 mg ml^−1^ OVA-647 in DPBS was injected into the cisterna magna as described previously^27^.

### Splenectomy

*Lta-*KO mice were anaesthetized by i.p. injection of ketamine (40–80 mg per kg) and xylazine (5–10 mg per kg). The skin over the left abdomen was sterilized with iodine solution, and a 0.5 cm incision was made with sterile scalpel. The spleen was exposed using smooth forceps, and vessels and attachments were ligated with absorbable 5-0 sutures before excision of the spleen. The midline fascial defect was closed with 4-0 absorbable sutures, and the skin was closed with interrupted 4-0 non-absorbable sutures.

### dCLN ligation and removal

dCLN ligation was performed as previously described^29,52^. In brief, mice were anaesthetized with ketamine–xylazine cocktail. An incision was made 5 mm above the clavicle. The sternocleidomastoid muscles were retracted, and afferent lymphatic vessels were ligated with a nylon suture. Sham-surgery group received the incision and muscle retraction but were not ligated. For lymphadenectomy, dCLNs were removed during this process. Mice were sutured and recovered on a heating pad until responsive. After surgery, mice were given analgesic and prophylactic antibiotics.

### Adoptive transfer

Spleens from donor mice were collected, and primary naive or whole CD4^+^ T cells and B cells were isolated using the EasySep Mouse Naive CD4^+^ T Cell Isolation Kit (StemCell Technologies, 19765), mouse CD4^+^ T cell isolation kit (StemCell Technologies, 19852) and the Mouse B Cell Isolation Kit (StemCell Technologies, 19854), respectively. A total of 2–5 × 10^5^ naive CD4^+^ T cells and 0.5–1 × 10^6^ B cells were resuspended in DPBS and intravenously injected through the retro-orbital route.

### Single-cell isolation and flow cytometry

For i.v. immune labelling, mice were anaesthetized and received 7.5 μg of CD45-BV750 (30-F11, BD Biosciences) intravenously through the retro-orbital route. Then, 3–5 min later, mice were euthanized. Mice were euthanized using a CO_2_ chamber and transcardially perfused with DPBS. Single-cell suspensions were prepared from each organ. Bones were collected and cleaned to remove attached soft tissues, and the dura was peeled from the skull. Single-cell suspensions from BM were obtained as previously described^4,5^. In brief, bones were mechanically dissociated by chopping with scissors in Roswell Park Memorial Institute (RPMI) 1640 medium (Gibco, 11875) containing 2% FBS.

Dura, chopped LNs, brain and brain tumour tissues were digested with a solution containing 1 mg ml^−1^ collagenase VIII (Sigma-Aldrich, C2139) and 0.5 mg ml^−1^ DNase I (Thermo Fisher Scientific, EN0521) at 37 °C for 30 min. Whole spleens or single-cell suspensions from various organs were passed through 70-μm strainers. Cell suspensions from brain and tumour tissues were centrifuged with 30% Percoll (Sigma-Aldrich, 17-0891-01) to remove myelin debris. Red blood cells were removed using ammonium–chloride–potassium (ACK) lysis buffer (Gibco, A1049201).

Single-cell suspensions were treated with anti-CD16/32 antibody (93, BioLegend) to block Fc receptors, then stained with antibodies targeting the following molecules: CD45 (30-F11, BioLegend), CD3 (17A2, BioLegend), CD4 (GK1.5, BD Biosciences), CD8a (53-6.7, BioLegend), CD11b (M1/70, BD Biosciences), CD11c (N418, BioLegend), I-A/I-E (M5/114.15.2, BioLegend), NK1.1 (PK136, Thermo Fisher Scientific), CD19 (1D3, BD Biosciences), CD45R/B220 (RA3-6B2, BD Biosciences), IgD (11-26c.2a, BioLegend), IgM (II/41, Thermo Fisher Scientific), IgA (mA-6E1, Thermo Fisher Scientific), IgG (Poly4053, BioLegend), CD138 (281-2, BD Biosciences), TACI (8F10, BD Biosciences), CD38 (90, BioLegend), FAS (SA367H8, BioLegend), CXCR5 (L138D7, BioLegend), CXCR3 (CXCR3-173, BioLegend), CD154 (MR1, BD Biosciences), CXCR4 (L276F12, BioLegend), ICOS (7E.17G9, Thermo Fisher Scientific), PD-1 (RMP1-30, BD Biosciences), CD44 (IM7, BD Biosciences), CD69 (H1.2F3, BD Biosciences), CD62L (MEL-14, BioLegend), IL-21R (4A9, BioLegend), Thy1.2 (53-2.1, BioLegend), IgMa (DS-1, BD Biosciences), IgMb (AF6-78, BD Biosciences), TCR Va2 (B20.1, BioLegend), TCR Vβ5.1/5.2 (MR9-4, BD Biosciences), CD31 (390, BioLegend), PDPN (8.1.1, BioLegend), CR1/2 (7E9, BioLegend), CD16/32 (93, BioLegend), CD25 (PC61, BioLegend), Ly6C (AL-21, BD Biosciences), Ly6G (1A8, BD Biosciences) and CD103 (2E7, BioLegend).

For intracellular staining, cells were fixed and permeabilized using the FoxP3 staining kit (BioLegend) according to the manufacturer’s instructions, and antibodies targeting T-bet (4B10, BioLegend), BCL6 (K112-91, BD Biosciences), IFNγ (XMG1.2, BioLegend), TNF (MP6-XT22, BioLegend), GZMB (NGZB, Thermo Fisher Scientific), IL-10 (JES5-16E3, BD Biosciences) and FOXP3 (FJK-16s, Thermo Fisher Scientific) were used. Live cells were gated using Zombie NIR fixable viability dye (BioLegend). For intracellular cytokine staining, single-cell suspensions were stimulated with PMA/ionomycin (Cell Stimulation Cocktail; 00-4970-93, Thermo Fisher Scientific) in the presence of Protein Transport Inhibitor Cocktail (00-4980-93, Thermo Fisher Scientific) for 4 h at 37 °C. Antibodies were diluted 1:200 for staining. All samples were acquired on an Aurora spectral flow cytometer (Cytek), and data were analysed using FlowJo software v.10.8.1 (TreeStar). The gating strategies and FMO control are shown in Supplementary Figs. 5 and 6.

### qPCR with reverse transcription

Tissues were isolated as described above. Total RNA was extracted from the brain and skull using TRIzol (Sigma-Aldrich, T9424), while RNA from dura was isolated using the RNAqueous-Micro Total RNA Isolation Kit (Thermo Fisher Scientific, AM1931) according to the manufacturer’s instructions. Purified RNA was reverse transcribed into cDNA using the iScript Reverse Transcription Supermix for quantitative PCR (qPCR) (Bio-Rad, 1708841). qPCR was performed on the QuantStudio 6 Flex system (Applied Biosystems).

The following primers were used to detect *eGFP*. Forward: 5′-AAGGGCATCGACTTCAAGG-3′; reverse: 5′-TGCTTGTCGGCCATGATATAG-3′. Transcript levels were normalized to *Actb*, and relative gene expression was calculated using the comparative ΔΔ*C*_t_ method.

### Immunofluorescence

Mice were perfused with Dulbecco’s phosphate-buffered saline (DPBS) followed by 4% paraformaldehyde (PFA) through intracardiac injection. The samples were then fixed in 4% PFA. Bone samples were decalcified in 5% EDTA solution at 4 °C for 3 days, with daily EDTA changes. For cryosectioning, samples were dehydrated in 30% sucrose solution at 4 °C for 3 days, then embedded in Fisher Healthcare Tissue-Plus OCT compound (Fisher Healthcare, 23-730-571). The blocks were frozen and sectioned at 20–50 μm thickness using a cryostat (Leica Biosystems).

For immunostaining, tissues were blocked in 0.2% Triton X-100 in DPBS with 5% goat or donkey serum, then stained with primary antibodies, including: Armenian hamster anti-mouse CD3e (145, Thermo Fisher Scientific), rabbit anti-mouse CD20 (SP32, Thermo Fisher Scientific), APC-conjugated mouse-anti-mouse IgMa (MA-69, BioLegend), rat anti-mouse BLIMP1 (6D3, Thermo Fisher Scientific), rabbit polyclonal anti-mouse S1PR2 (Proteintech), Alexa Fluor 647-conjugated rat anti-mouse PD-1 (29 F.1A12, BioLegend), Alexa Fluor 594-conjugated rat anti-CD4 (GK1.5, BioLegend), Alexa Fluor 488-conjugated rat anti-mouse CD20 (SA275A11, BioLegend), polyclonal goat anti-mouse CXCL13 (R&D Systems), polyclonal goat anti-mouse BAFF (R&D Systems), CD35 chimeric recombinant rabbit antibody (8C12, Thermo Fisher Scientific), rat anti-mouse FDC (FDC-M1, BD Biosciences), rabbit polyclonal anti-mouse BCL6 (Thermo Fisher Scientific) and eFluor 660-conjugated rat anti-mouse B220 (RA3-6B2, Thermo Fisher Scientific), polyclonal anti-GFP (Thermo Fisher Scientific, A10262). Secondary antibodies included: Alexa Fluor 488-conjugated goat anti-Armenian hamster IgG (Jackson Laboratory, 127-545-099), Alexa Fluor 594-conjugated donkey anti-rabbit IgG (Thermo Fisher Scientific, A32754), Alexa Fluor 647-conjugated donkey anti-goat IgG (Thermo Fisher Scientific, A32849), Alexa Fluor 647-conjugated donkey anti-rat IgG (Thermo Fisher Scientific, A78947), Alexa 488-conjugated donkey anti-rat IgG (Thermo Fisher Scientific, A48269), Alexa Fluor 647-conjugated donkey anti-rabbit IgG (Thermo Fisher Scientific, A32795TR), Alexa Fluor 488-conjugated donkey anti-rabbit IgG (Thermo Fisher Scientific, A-21206).

For in vivo labelling, the following antibodies were injected intravenously 40 min before perfusion: 30–100 μg of Alexa Fluor 488-conjugated anti-mouse CD20 (SA275A11, BioLegend), Alexa Fluor 594-conjugated anti-mouse I-A/I-E (M5/114.15.2, BioLegend), coralite594-conjugated anti-mouse Thy1.2 (30-H12, Thermo Fisher Scientific), Alexa Fluor 594-conjugated anti-mouse CD31 (390, BioLegend) and eFluor 660-conjugated anti-mouse B220 (RA3-6B2, Thermo Fisher Scientific), Alexa Fluor 594-conjugated anti-mouse CD4 (GK1.5, BioLegend) and Alexa Fluor 647-conjugated anti-mouse CD4 (GK1.5, BioLegend). Nuclei were stained with 4′,6-diamidino-2-phenylindole (DAPI, Sigma-Aldrich, D9542).

For whole-mount staining, the ADAPT-3D protocol was used for tissue clearing^53^. Whole-mount staining of the dura was performed as previously described^27^. Images were acquired using the wide-field microscopy (Olympus SLIDEVIEW VS200) or Stellaris confocal microscope (Leica) with ×10, ×20 or ×40 objectives with Leica Application Suite v.4.2.1.23810 and Nikon Elements v.5.2.0 and analysed with Fiji software v.2.14.0/1.54j. The colocalization plugin in Fiji was used to identify co-localized cells. Cell counting and density calculation were performed with QuPath-0.6.0 software^54^. The intensity and coverage of fluorescent proteins were analysed using Fiji software. The background fluorescence was determined using negative controls, such as non-fluorescent samples or regions. Values were not normalized. Representative single-plane images of whole mounted skulls are provided in Supplementary Fig. 7.

### Single-cell transcriptomic analysis

Public data were downloaded from the Gene Expression Omnibus (GEO) under accession numbers GSE184766 (ref. ^4^) and GSE233304 (ref. ^8^). For T and B cell scRNA-seq analysis, single-cell suspensions from the skull and sternum of healthy 8-week-old C57BL/6J male mice were prepared as described above. Single-cell suspensions were treated with anti-CD16/32 (2.4G2) to block Fc receptors. For T cell isolation, cells were stained with PerCP/Cy5.5-conjugated anti-CD45 (30-F11, BioLegend), PE/Cy7-conjugated anti-CD11b (M1/70, BD Biosciences), PE-conjugated anti-Thy1.2 (30-H12, Thermo Fisher Scientific), eFluor 660-conjugated anti-B220 (RA3-6B2, Thermo Fisher Scientific), FITC-conjugated anti-CD4 (RM4-4, BD Biosciences) and Alexa Fluor 488-conjugated CD8a (4SM16, Thermo Fisher Scientific). Dead cells were labelled with DAPI. Live CD45^+^B220^−^CD11b^−^Thy1.2^+^CD4/CD8^+^ cells were sorted. For B cell and ASC isolation, cells were stained with PerCP/Cy5.5-conjugated anti-CD45 (30-F11, BioLegend), FITC-conjugated anti-CD3 (17A2, Thermo Fisher Scientific), Alexa Fluor 700-conjugated anti-CD19 (6D5, BioLegend), APC-conjugated anti-CD138 (281-2, Thermo Fisher Scientific), PE-conjugated anti-TACI (8F10, BD Biosciences) and Pacific Blue-conjugated anti-IgD (11-26 c.2a, BioLegend). Dead cells were labelled with Zombie-NIR viability dye (BioLegend). Live CD45^+^CD3^−^CD138^−^CD19^+^ B cells and live CD45^+^IgD^−^CD3^−^CD138^+^TACI^+^ ASCs were isolated using the FACSAria II (BD Biosciences) system. For GC B cell scRNA-seq, single-cell suspensions from skull and tumour tissues were prepared as described above, 14 days after tumour injection. GC B cells were isolated using the GC B cell (PNA) microbead kit (Miltenyi Biotec). Dead cells were labelled with DAPI, and DAPI^−^ live cells were sorted using the FACSAria III (BD Biosciences) system.

Cells were loaded onto a 10x Genomics Chromium platform for gel beads-in-emulsions. Single Cell 5′ Library & Gel Bead Kit and Mouse B Cell Chromium V(D)J Reagents Kits were used to generate cDNA libraries targeting BCRs and gene expression. Libraries were sequenced using the Illumina NovaSeq 6000 system. Data were aligned to the mm10 genome using the CellRanger software pipeline (10x Genomics; v.1.1.0). Matrices were loaded into Seurat v.4 or v.5 for analysis^55^, and R v.4.4.0 was used for statistical analyses. Cells with unique feature counts <200 or >6,000 (for T cell data), <200 or >7,500 (for B cell data), and <200 or >8,000 (for GC B cell data) with >20% (for T and B cell data) and >5% (skull) or >10% (tumour tissue) (for GC B cell data) mitochondrial gene expression were excluded for quality control. Doublets were also removed. Filtered and normalized data were analysed with the Seurat pipeline. Objects were merged and principal component analysis (PCA) and UMAP analysis were conducted using an elbow plot. Shared nearest neighbour (SNN) clustering was optimized with the Louvain algorithm in the FindClusters function. V(D)J receptor annotation data were generated with the CellRanger vdj command. Filtered contig CSV files were imported into R, and productive barcodes were filtered. The scRepertoire pipeline was used for scBCR/RNA-seq data analysis^56^. Cluster markers were identified using the FindMarkers function, and cluster identities were determined manually. The Nebulosa package was used to visualize gene expression density^57^. Somatic hypermutation frequencies were calculated using BCR sequence data processed with the Immcantation pipeline (https://immcantation.readthedocs.io/en/stable/)^58,59^ and the IgBLAST and IMGT germline sequence databases. The Change-O package was used to process V(D)J annotations in BCR data files, and clonal family sizes were calculated using the Alakazam package. BCR mutation frequencies of IGHV were calculated using the SHazaM package.

### OVA measurement

AAV-eGFP-, AAV-OVA-infected brains were chopped, homogenized with 70 μm strainer. After centrifugation, supernatants were collected and stored at −80 °C. CSF from AAV-eGFP- or AAV-HEL-OVA-infected mice was collected as described before^52^. OVA was measured by enzyme-linked immunosorbent assay (ELISA) using the sandwich OVA ELISA kit (LS Bio, LS-F9540-1) according to the manufacturer’s instructions. BioTek Gen5 software v.3.11 (Agilent) was used.

### Statistical analysis

All data are presented as the mean ± s.e.m. Differences between two experimental groups were analysed using a two-tailed paired or unpaired Student’s *t*-test and Mann–Whitney *U*-test. For comparisons involving more than two groups, one-way ANOVA with Tukey’s multiple comparison test was used. For paired samples, Dunnett’s correction was applied. Two-way ANOVA with Sidak’s correction was also used, with paired comparisons used when samples were paired. Survival data were analysed using the log-rank test, and if multiple comparisons were required, the Holm–Sidak method was used for correction. All data are representative of results from at least two independent experiments. scBCR/RNA-seq data were analysed and visualized using R software (v.4.4.0) with RStudio, and all other data were analysed and plotted with GraphPad Prism (v.10.3.1).

### Reporting summary

Further information on research design is available in the Nature Portfolio Reporting Summary linked to this article.

## Online content

Any methods, additional references, Nature Portfolio reporting summaries, source data, extended data, supplementary information, acknowledgements, peer review information; details of author contributions and competing interests; and statements of data and code availability are available at https://doi.org/10.1038/s41586-026-10951-4.

## Supplementary information

Supplementary Figs. 1–7 Suppplementary Figs. 1–7.

Reporting Summary

## Source data

Source Data Fig. 1

Source Data Fig. 2

Source Data Fig. 3

Source Data Fig. 4

Source Data Fig. 5

Source Data Extended Data Fig. 1

Source Data Extended Data Fig. 2

Source Data Extended Data Fig. 3

Source Data Extended Data Fig. 4

Source Data Extended Data Fig. 5

Source Data Extended Data Fig. 6

Source Data Extended Data Fig. 7

Source Data Extended Data Fig. 8

Source Data Extended Data Fig. 9

Source Data Extended Data Fig. 10

Source Data Extended Data Fig. 11

Source Data Extended Data Fig. 12

## Data Availability

GSE184766 (ref. ^4^) (cerebrospinal fluid regulates skull bone marrow niches via direct access through dural channels) and GSE233304 (ref. ^8^) (cranioencephalic functional lymphoid units in glioblastoma) are publicly available. All original raw scRNA-seq data are available at the GEO under accession numbers (GSE309632, GSE309634 and GSE313969). All data generated in this study are available on request from the corresponding authors. Source data are provided with this paper.

## Code availability

No custom code or algorithms were developed for this study. All sequencing and imaging analyses were performed using standard, publicly available software packages and established analysis pipelines, as described in the Methods. Any additional information is available from the corresponding authors on reasonable request.
