# Supplementary material for: Functional role of skull lymphoid structures in CNS immunosurveillance
Source: Nature. 2026 Aug 19;658(8134):196–204. doi: 10.1038/s41586-026-10951-4 (PMC13626955; doi:10.1038/s41586-026-10951-4)
Supplement: Supplementary file 1 [file 41586_2026_10951_MOESM1_ESM.pdf]

nature portfolio

https://doi.org/10.1038/s41586-026-10951-4

Supplementary information

Functional role of skull lymphoid structures in CNS immunosurveillance

In the format provided by the authors and unedited

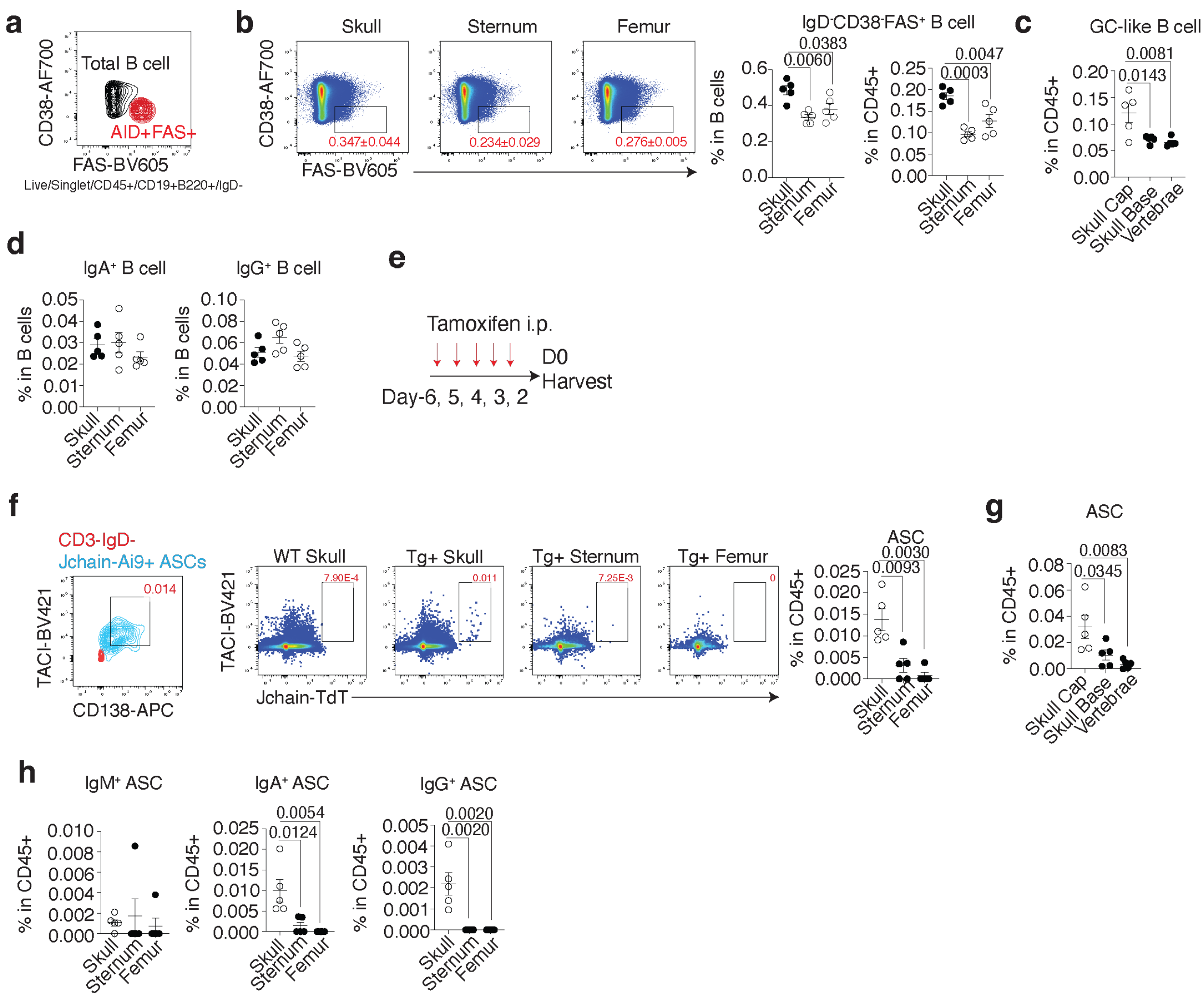

Supplementary Figure 1. Skull BM B cell activation and differentiation compared with other BM sites.

a. Representative flow cytometry plot of GC-like B cells (gray, total B cells; red, GC-like CD38$^-$ IgD$^-$FAS$^+$AID$^+$ cells from Fig. 2e).

b-c. Frequency of IgD$^-$FAS$^+$CD38$^-$ GC-like B cells among B cells and total immune cells comparing with peripheral BMs (**b**) and CNS-associated BMs (**c**). n=5 mice per group.

d. Frequency of IgA$^+$ and IgG$^+$ cells in B cells. n=5 of mice were used.

e. Experimental scheme using Jchain-CreERT2::Ai9 mice. i.p., intraperitoneal injection.

f. Overlapped FACS plots between total $CD3^{-}IgD^{-}$ cells and Jchain-$Ai9^{+}$ ASCs (left) and frequency of $Jchain^{+}TACI^{+}$ ASCs among immune cells (right). n=5 mice per group.

g. The frequency of ASCs ($CD138^{+}TACI^{+}$) among CNS-associated BMs. n=5 mice per group.

h. Subsets of ASCs ($Jchain^{+}TACI^{+}$) among immune cells. n=5 of mice were used.

Data are represented as mean ± s.e.m. One-way ANOVA with Dunnett's multiple test was used. *P* values are shown as the exact values.

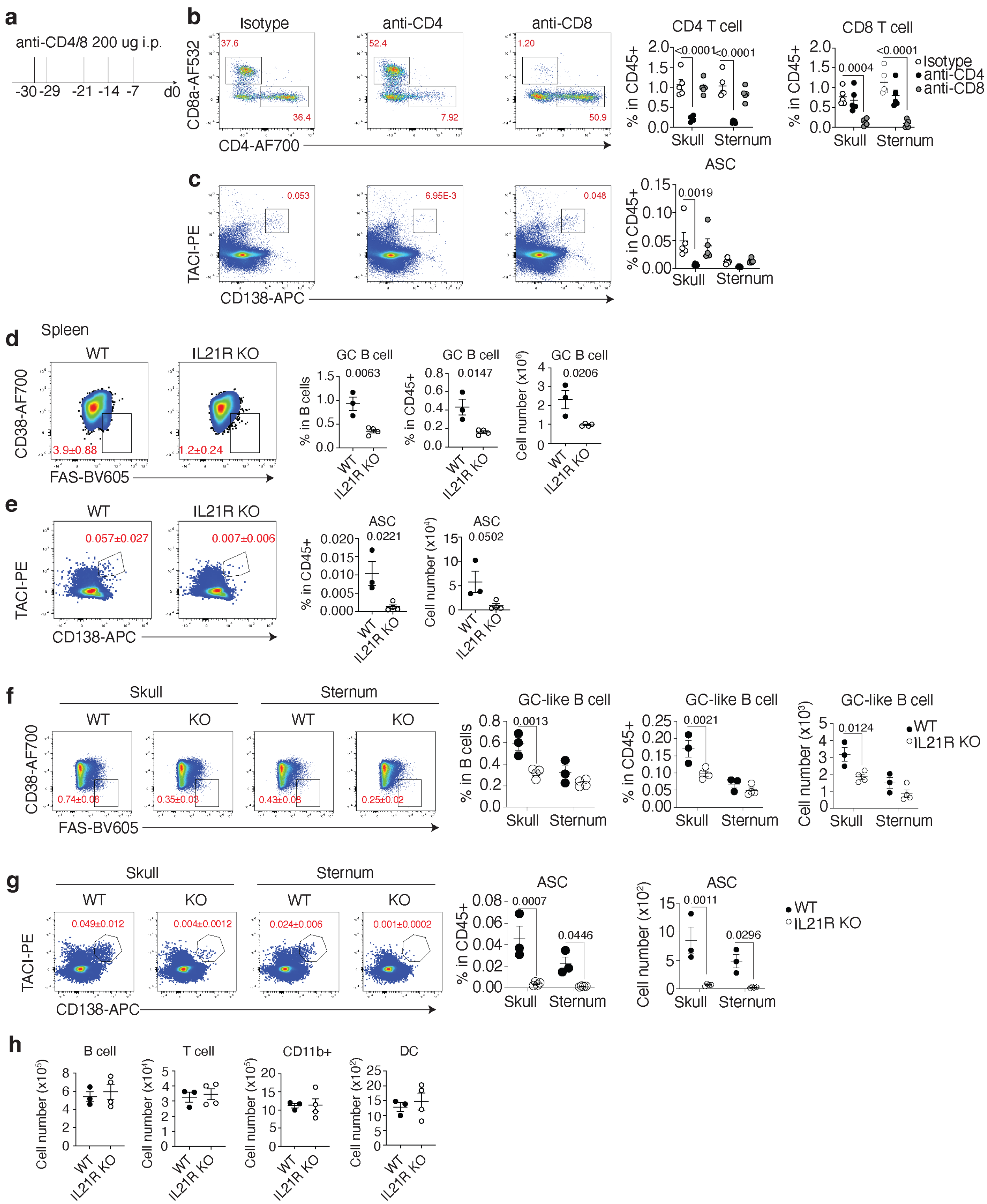

Supplementary Figure 2. Tfh cells support activation and differentiation of skull BM B cells.

a. Experimental scheme of T cell depletion.

b. Flow cytometry analysis confirming CD4 and CD8 T cell depletion (gating plot, left; dot graphs, right). n=5 mice per group.

c. Flow cytometry analysis of ASCs after T cell depletion. n=5 of mice per group were used.

d-e. Flow cytometry analysis comparing GC-like B cells (**d**), ASCs (**e**) from spleen between WT and IL-21R KO mice. n = 3 (WT) and n = 4 (IL21R KO) mice.

f-g. Flow cytometry analysis comparing GC-like B cells (**f**) and ASCs (**g**) in the skull and sternum bone marrow (BM) from wild-type (WT) and IL21R knockout (KO) mice. n = 3 (WT) and n = 4 (IL21R KO) mice.

h. Cell numbers of skull immune cells. n = 3 (WT) and n = 4 (IL21R KO) mice.

Data represent mean ± s.e.m. Two-way ANOVA with Sidak's multiple comparisons (**b, c, f, g**) and two-tailed unpaired Student's *t*-test (**d, e, h**) were used. *P* values are shown as the exact values.

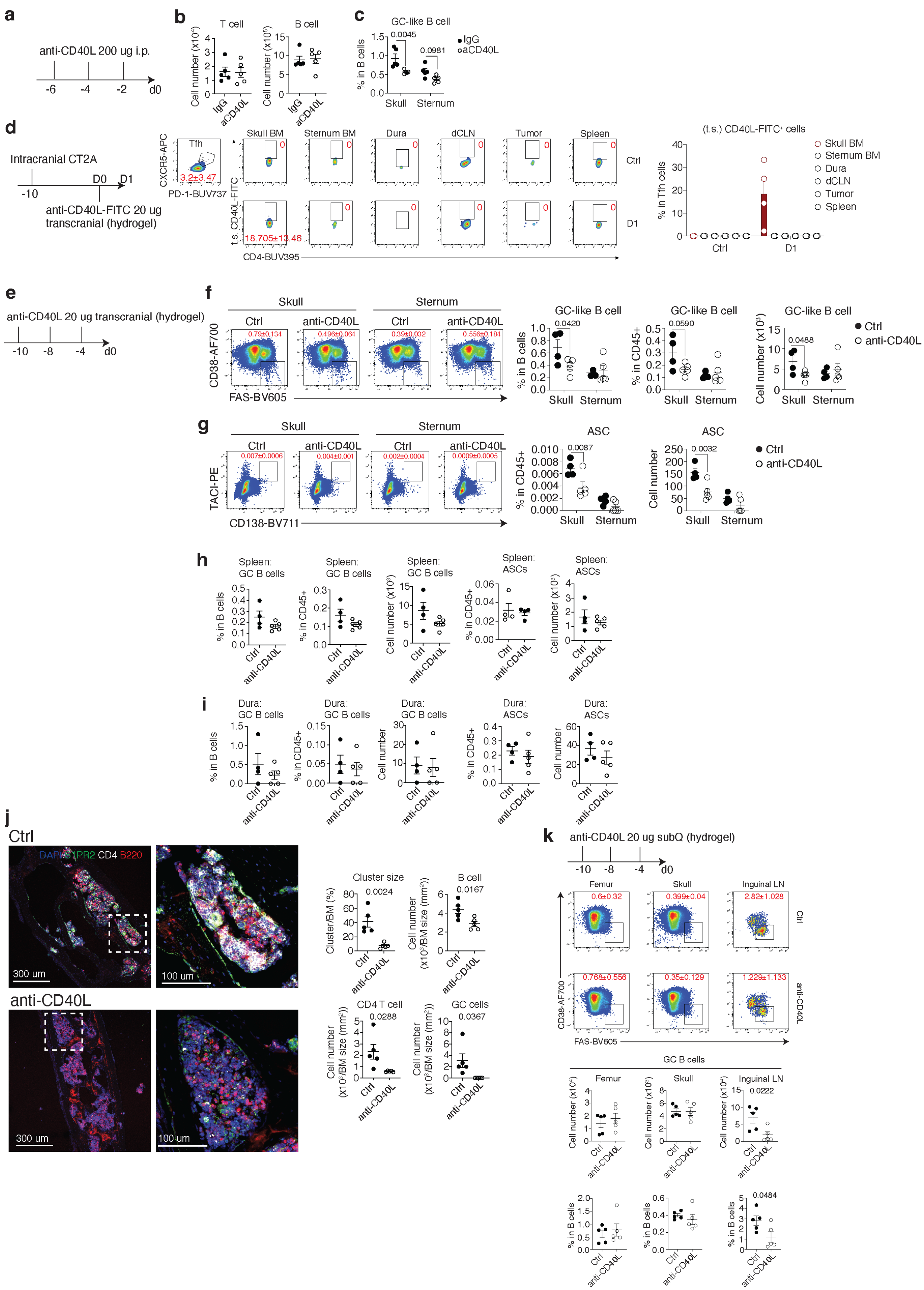

a
anti-CD40L 200 ug i.p.
-6 -4 -2 d0
b
T cell
B cell
c
GC-like B cell
IgG
aCD40L
Skull Sternum
d
Intracranial CT2A
anti-CD40L-FITC 20 ug transcranial (hydrogel)
Skull BM
Sternum BM
Dura
dCLN
Tumor
Spleen
Ctrl
D1
(t.s.) CD40L-FITC⁺ cells
e
anti-CD40L 20 ug transcranial (hydrogel)
f
GC-like B cell
Ctrl
anti-CD40L
CD38-AF700
FAS-BV605
g
ASC
TACI-PE
CD138-BV711
h
Spleen: GC B cells
Spleen: ASCs
i
Dura: GC B cells
Dura: ASCs
j
Ctrl
anti-CD40L
Cluster size
B cell
CD4 T cell
GC cells
k
anti-CD40L 20 ug subQ (hydrogel)
Femur
Skull
Inguinal LN
GC B cells

Supplementary Figure 3. CD40-CD40L axis is required for B cell activation by Tfh cells.

a. Experimental scheme of systemic CD40L blockade.

b. Numbers of T and B cells in the skull BM. n = 5 mice per group.

c. Percentage of GC-like B cells in the skull and sternum BM. n=5 of mice per group were used.

d. Flow cytometry analysis identifying FITC-conjugated anti-CD40L antibody-bound Tfh cells after local injection with hydrogel. Experimental scheme (left), FACS plots (middle), and bar graphs (right). n = 4 mice per group.

e. Experimental scheme for local injection of anti-CD40L antibody.

f-g. Flow cytometry analysis comparing GC-like B cells (**f**) and ASCs (**g**) in the skull and sternum BM from control (ctrl) and anti-CD40L antibody-treated mice. n = 4 (ctrl) and n = 5 (anti-CD40L).

h-i. Splenic (**h**) and dura (**i**) GC B cells and ASCs after local anti-CD40L treatment. n = 4 (ctrl) and n = 5 (anti-CD40L).

j. GC B cells and CD4 T cells in the posterior skull BM coronal sections (left) and quantification (right). n=5 of mice per group were used.

k. Experimental scheme of peripheral anti-CD40L injection (upper), representative FACS plots (middle), and quantifications (below). n=5 of mice per group were used.

Data represent mean ± s.e.m. Two-way ANOVA with Sidak's multiple comparisons (**c, f, g**) and two-tailed unpaired Student's *t*-test (**b, h-k**) were used. *P* values are shown as the exact values.

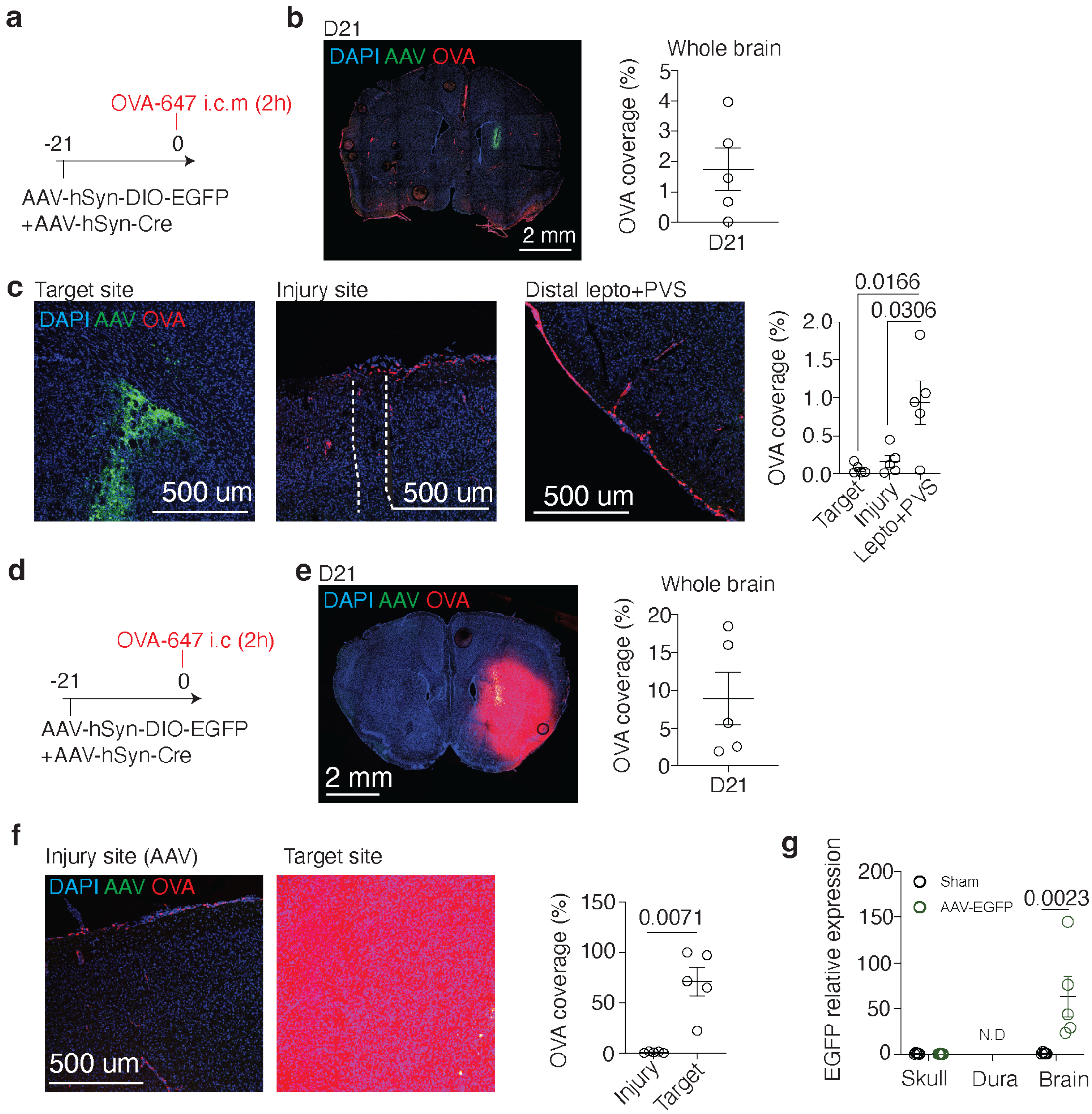

Supplementary Fig. 4. Antigen drainage is not mediated by surgery-induced injury

a-c. After 21 days of intracranial AAV-EGFP injection, OVA-647 tracers were injected into the cisterna magna. After 2 hours of i.c.m. OVA injection, tissues were harvested and analyzed with IHC (**a**). Coronal section of brain (**b**). Zoomed in images for specific regions (left) and quantifications of OVA coverage (right) (**c**). n=5 mice per group were used.

d-f. After 21 days of intracranial AAV-EGFP injection, OVA-647 tracers were intracranially injected. After 2 hours of OVA injection, tissues were harvested and analyzed with IHC (**d**). Coronal section of brain (**e**) and specific regions (**f**) with quantifications. n=5 mice per group were used.

g. *Egfp* mRNA expression levels in different tissues compared to *Actb* genes after 4 weeks of sham surgery or AAV-EGFP intracranial injection. n=5 of mice per group were used.

Data are represented as mean ± s.e.m. Two-tailed paired Student's t-test (**f**), one-way ANOVA with Tukey's multiple comparison (**c**), and two-way ANOVA with Sidak's multiple comparison (**g**) were used.

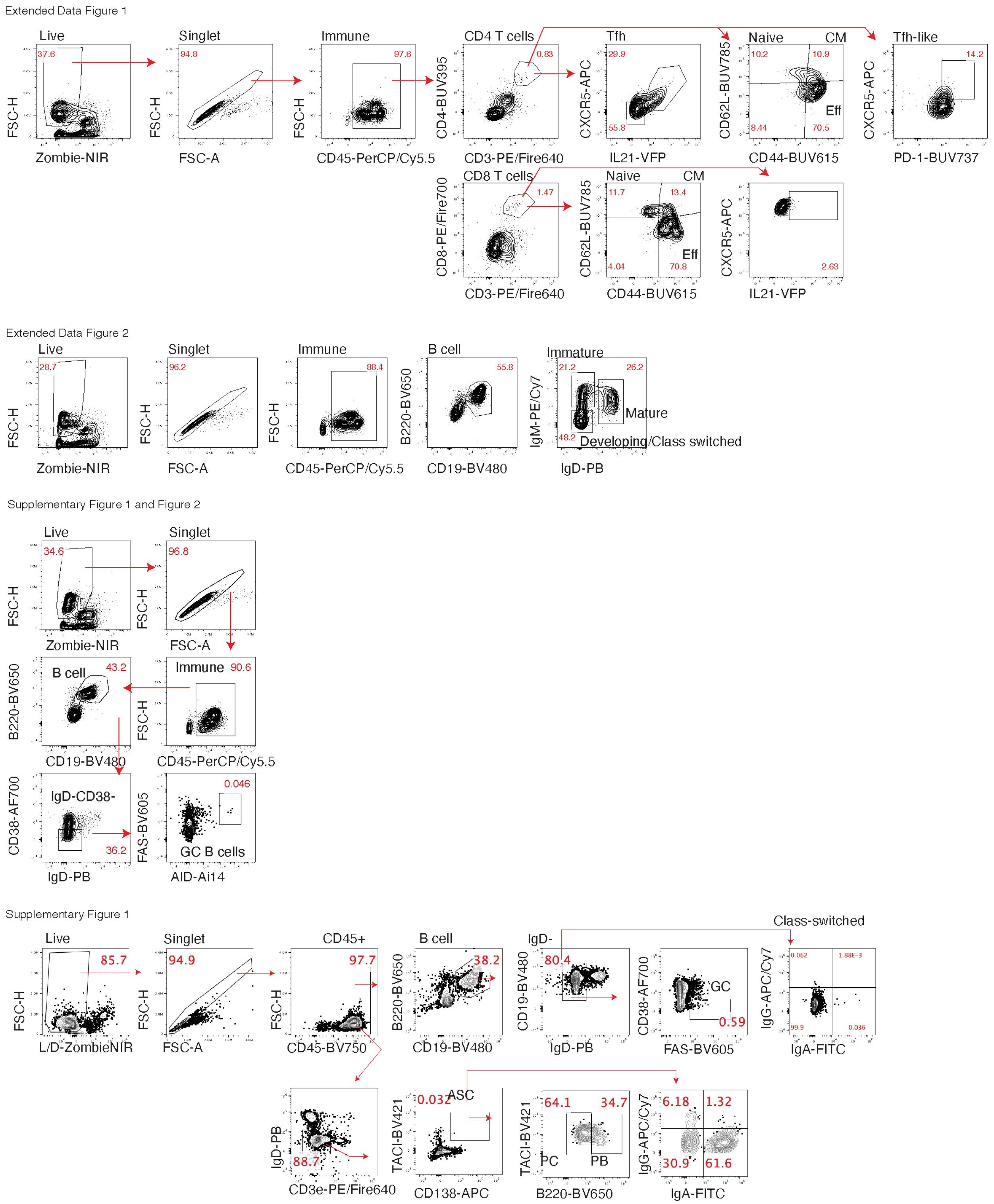

Supplementary Figure 5. Gating strategy for Extended Data Figure 1, 2, Supplementary Figure 1, and Figure 2.

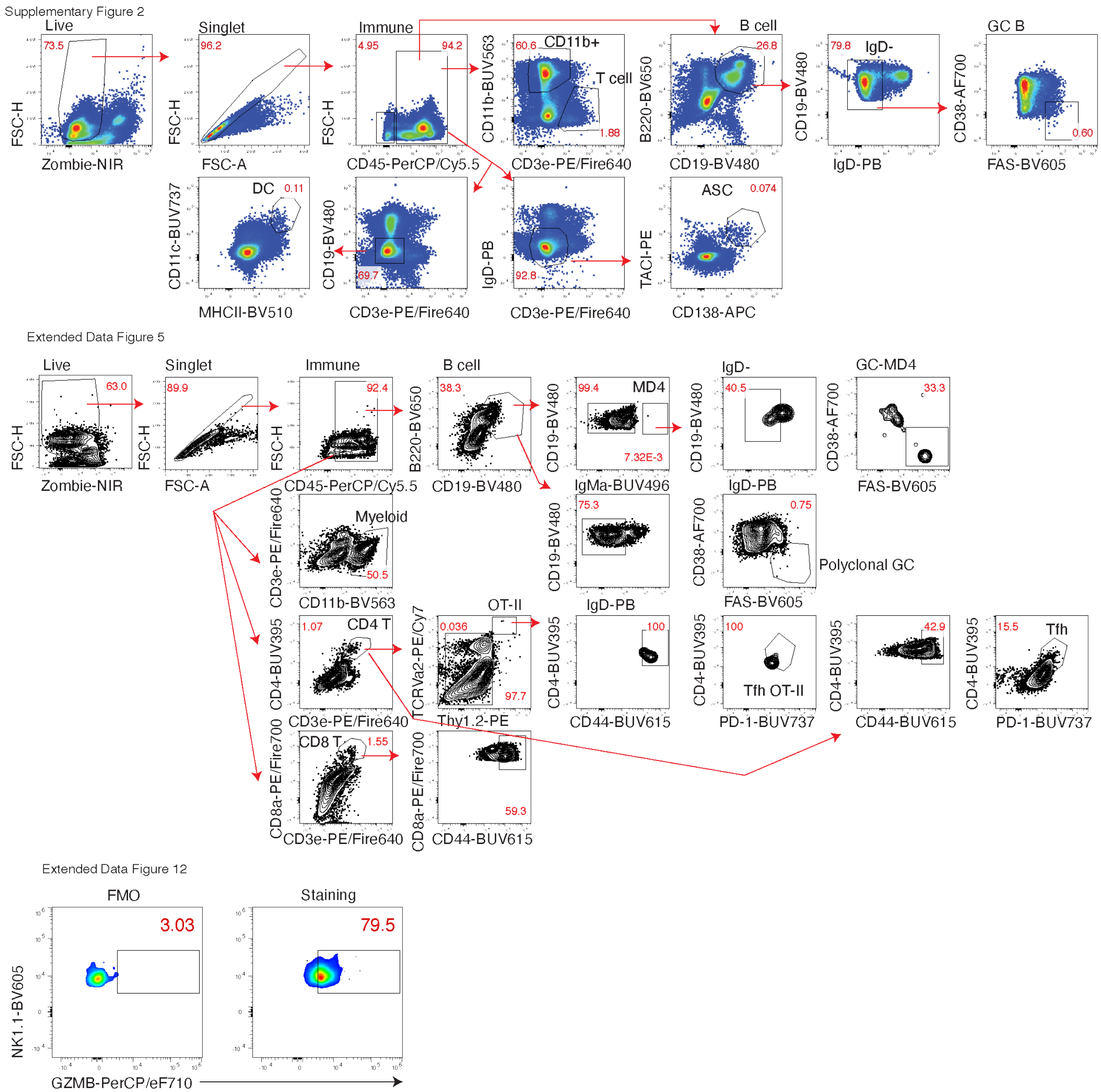

Supplementary Figure 6. Gating strategies and FMO control for flow cytometry analyses.

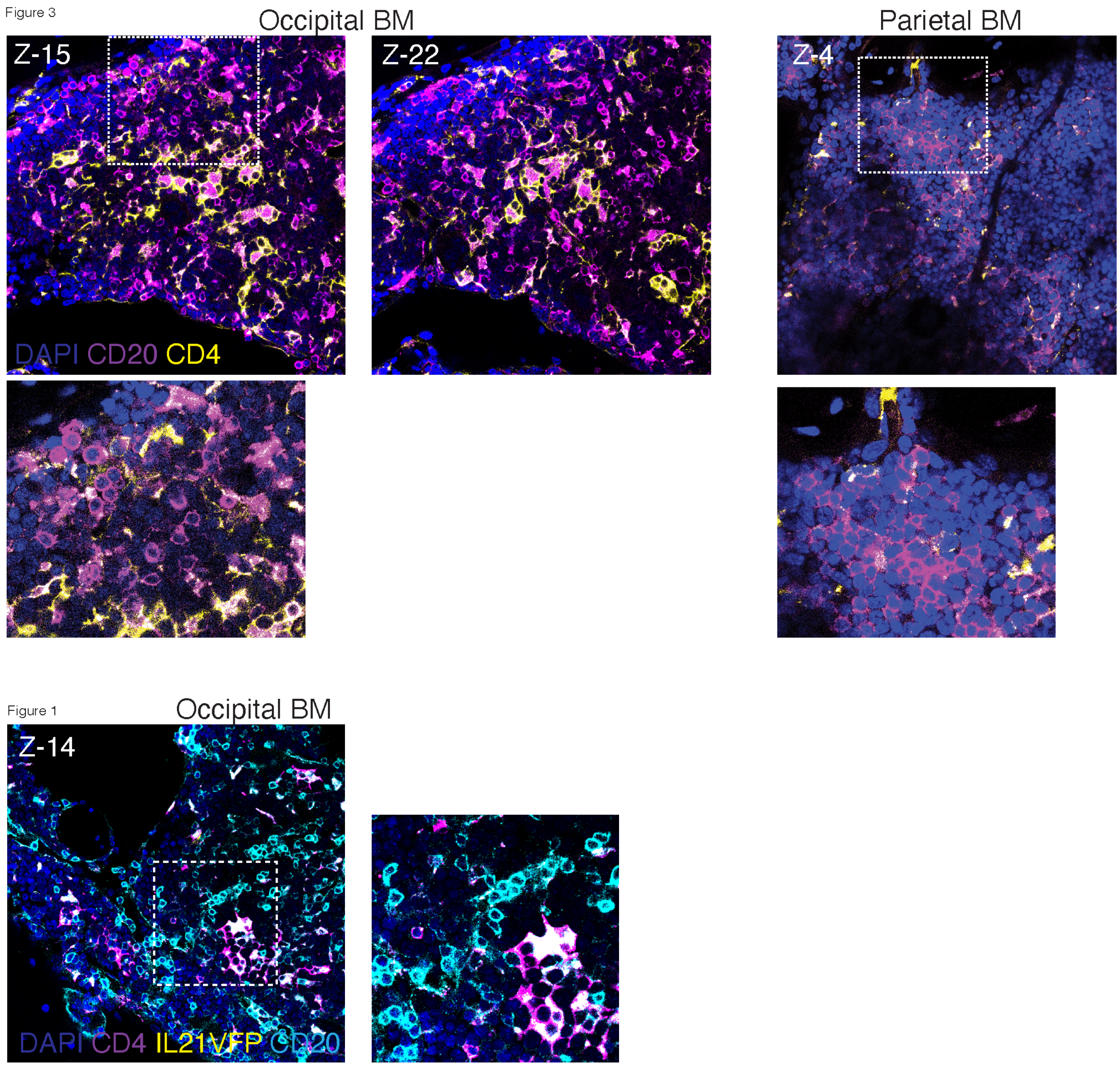

Supplementary Figure 7. Single plane images of skull whole mount images for Figure 1 and 3.
