# Supplementary material for: Functional role of skull lymphoid structures in CNS immunosurveillance
Source: Nature. 2026 Aug 19;658(8134):196–204. doi: 10.1038/s41586-026-10951-4 (PMC13626955; doi:10.1038/s41586-026-10951-4)
Supplement: Supplementary file 2 [file 41586_2026_10951_MOESM2_ESM.pdf]

nature portfolio

Corresponding author(s): Jonathan Kipnis

Last updated by author(s): YYYY-MM-DD

Reporting Summary

Nature Portfolio wishes to improve the reproducibility of the work that we publish. This form provides structure for consistency and transparency in reporting. For further information on Nature Portfolio policies, see our Editorial Policies and the Editorial Policy Checklist.

Statistics

For all statistical analyses, confirm that the following items are present in the figure legend, table legend, main text, or Methods section.

n/a	Confirmed	
☐	☒	The exact sample size (*n*) for each experimental group/condition, given as a discrete number and unit of measurement
☐	☒	A statement on whether measurements were taken from distinct samples or whether the same sample was measured repeatedly
☐	☒	The statistical test(s) used AND whether they are one- or two-sided<br>*Only common tests should be described solely by name; describe more complex techniques in the Methods section.*
☒	☐	A description of all covariates tested
☐	☒	A description of any assumptions or corrections, such as tests of normality and adjustment for multiple comparisons
☐	☒	A full description of the statistical parameters including central tendency (e.g. means) or other basic estimates (e.g. regression coefficient) AND variation (e.g. standard deviation) or associated estimates of uncertainty (e.g. confidence intervals)
☐	☒	For null hypothesis testing, the test statistic (e.g. *F*, *t*, *r*) with confidence intervals, effect sizes, degrees of freedom and *P* value noted<br>*Give P values as exact values whenever suitable.*
☒	☐	For Bayesian analysis, information on the choice of priors and Markov chain Monte Carlo settings
☒	☐	For hierarchical and complex designs, identification of the appropriate level for tests and full reporting of outcomes
☒	☐	Estimates of effect sizes (e.g. Cohen's *d*, Pearson's *r*), indicating how they were calculated

Our web collection on statistics for biologists contains articles on many of the points above.

Software and code

Policy information about availability of computer code

Data collection

The following softwares were used for data collection in this study:
- Illumina NovaSeq 6000
- CYTEK SpectroFlo
- Cellranger_1.1.0
- Leica Application Suite v 4.2.1.23810
- Nikon Elements v 5.2.0
- BioTek Gen5 3.11
- QuantStudio 6 Flex System

Data analysis

The following softwares were used for data analysis in this study:
- Prism v10.3.1.
- FlowJo v10.8.1
- R v.4.4.0
- Rstudio
- 10X genomics Cellranger software pipeline
- Seurat v4, 5
- FIJI Image processing software - v. 2.14.0/1.54j
- Gen5 software v 3.11
- QuPath-0.6.0
- Immcantation pipeline

- scRepertoire 2.4.0
- Nebulosa 3.21

For manuscripts utilizing custom algorithms or software that are central to the research but not yet described in published literature, software must be made available to editors and reviewers. We strongly encourage code deposition in a community repository (e.g. GitHub). See the Nature Portfolio guidelines for submitting code & software for further information.

Data

Policy information about availability of data

All manuscripts must include a data availability statement. This statement should provide the following information, where applicable:

- Accession codes, unique identifiers, or web links for publicly available datasets
- A description of any restrictions on data availability
- For clinical datasets or third party data, please ensure that the statement adheres to our policy

GSE1847664 (Title: Cerebrospinal fluid regulates skull bone marrow niches via direct access through dural channels; https://www.ncbi.nlm.nih.gov/geo/query/acc.cgi?acc=GSE184766) and GSE2333048 (Title: Cranioencephalic functional lymphoid units in glioblastoma; https://www.ncbi.nlm.nih.gov/geo/query/acc.cgi?acc=GSE233304) are publicly available. All original raw single-cell sequencing data are available at the Gene Expression Omnibus (GEO) under the accession number (GSE309632, GSE309634, and GSE313969). All data generated in this study are available upon request from the corresponding authors. Source data are provided with this paper.

Research involving human participants, their data, or biological material

Policy information about studies with human participants or human data. See also policy information about sex, gender (identity/presentation), and sexual orientation and race, ethnicity and racism.

Reporting on sex and gender	N/A
Reporting on race, ethnicity, or other socially relevant groupings	N/A
Population characteristics	N/A
Recruitment	N/A
Ethics oversight	N/A

Note that full information on the approval of the study protocol must also be provided in the manuscript.

Field-specific reporting

Please select the one below that is the best fit for your research. If you are not sure, read the appropriate sections before making your selection.

☒ Life sciences ☐ Behavioural & social sciences ☐ Ecological, evolutionary & environmental sciences

For a reference copy of the document with all sections, see nature.com/documents/nr-reporting-summary-flat.pdf

Life sciences study design

All studies must disclose on these points even when the disclosure is negative.

Sample size	Statistical methods were not used to recalculate or predetermine study sizes. However, it was based on similar experiments previously published (Cugurra, Mamuladze, Rustenhoven et al., Science (2021); Rustenhoven et al. Cell (2021); Mazzitelli and Smyth et al. Nature Neuroscience (2022); Park et al. Nature Immunology (2021).
Data exclusions	No data were excluded for analysis.
Replication	All experiments were replicated at least two independent experiments with similar results. All representative images are representative of at least 3 animals.
Randomization	Mice were allocated randomly to cages with no more than 5 mice in one cage. Cages were randomly assigned to experimental groups. No covariates were present.
Blinding	No blinding was performed to prevent cross-contamination between groups (e.g. genotypes).

Reporting for specific materials, systems and methods

We require information from authors about some types of materials, experimental systems and methods used in many studies. Here, indicate whether each material, system or method listed is relevant to your study. If you are not sure if a list item applies to your research, read the appropriate section before selecting a response.

Materials & experimental systems

n/a	Involved in the study
☐	☒ Antibodies
☐	☒ Eukaryotic cell lines
☒	☐ Palaeontology and archaeology
☐	☒ Animals and other organisms
☒	☐ Clinical data
☒	☐ Dual use research of concern
☒	☐ Plants

Methods

n/a	Involved in the study
☒	☐ ChIP-seq
☐	☒ Flow cytometry
☒	☐ MRI-based neuroimaging

Antibodies

Antibodies used

Flow cytometry: CD16/32 (clone:93, 10302, BioLegend), CD16/32-APC/Cy7 (clone: 93, 101328, BioLegend), CD45-PerCP/Cy5.5 (clone: 30-F11, 103132, BioLegend), CD45-BV750 (clone: 30-F11, 746947, BD Biosciences), CD3-FITC (clone: 17A2, 11-0032-82, Thermo Scientific), CD3-PE/Fire640 (clone: 17A2, 100270, BioLegend), CD4-BUV395 (clone: GK1.5, 563790, BD Biosciences), CD4-FITC (clone RM4-4, 553055, BD Biosciences), CD4-Alexa Fluor 700 (clone: RM4-5, 557956, BD Biosciences), CD8a-Alexa Fluor 488 (clone: 4SM16, 740029TP488, Thermo Scientific), CD8a-PE/Fire700 (clone: 53-6.7, 100792, BioLegend), CD8a-Alexa Fluor 532 (clone: 53-6.7, 58-0081-80, Thermo Scientific), CD11b-BUV563 (clone: M1/70, 741242, BD Biosciences), CD11b-PE/Cy7 (clone: M1/70, 552850, BD Biosciences), CD11c-BUV737 (clone: N418, 749039, BD Biosciences), CD11c-FITC (clone: N418, 117306, BioLegend), I-A/I-E-BV510 (clone: M5/114.15.2, 107635, Biolegend), NK1.1-BV605 (clone: PK136, 108740, BioLegend), NK1.1-PE (clone: PK136, 553165, BD Biosciences), CD19-Alexa Fluor 700 (clone: 6D5, 115528, BioLegend), CD19-BV480 (clone: 1D3, 566107, BD Biosciences), B220-BV650 (clone: RA3-6B2, 563893, BD Biosciences), B220-BUV496 (clone: RA3-6B2, 612950, BD Biosciences), IgD-Pacific Blue (clone: 11-26c.2a, 405712, BioLegend), IgM-PE/Cy7 (clone: II/41, 25-5790-82, Thermo Scientific), IgA-FITC (clone: mA-6E1, 50-112-2116, Thermo Scientific), IgA-PE (clone: mA-6E1, 50-107-73, Thermo Scientific), IgG-APC (clone: Poly4053, 405308, BioLegend), IgG-APC/Cy7 (clone: Poly4053, 405316, BioLegend), CD138-APC (clone: 281-2, 558626, BD Biosciences), CD138-PerCP/Cy5.5 (clone: 281-2, 142509, BioLegend), CD138-BV711 (clone: 281-2, 563193, BD Biosciences), TACI-PE (clone: 8F10, 558410, BD Biosciences), TACI-BV421 (clone: 8F10, 742840, BD Biosciences), CD38-Alexa Fluor 700 (clone: 90, 102742, BioLegend), FAS-BV605 (clone: SA367H8, 152612, BioLegend), CXCR5-APC (clone: L138D7, 145506, BioLegend), CXCR3-PE (clone: CXCR3-173, 126506, BioLegend), CD154-BV650 (clone: MR1, 740480, BD Biosciences), CXCR4-BV421 (clone: L276F12, 146511, BioLegend), ICOS-PE/Cy7 (clone: 7E.17G9, 25-9942-82, Thermo Scientific), PD-1-BUV737 (clone: RMP1-30, 568363, BD Biosciences), CD44-BUV615 (clone: IM7, 751414, BD Biosciences), CD62L-BV785 (clone: MEL-14, 104440, BioLegend), IL21R-PE/Cy5 (clone: 4A9, 131908, BioLegend), Thy1.2-PE (clone: 30-H12, 12-0903-82, Thermo Scientific), IgMa-BUV496 (clone: DS-1, 750192, BD Biosciences), IgMb-BUV805 (clone: AF6-78, 749405, BD Biosciences), TCR Va2-PE/Cy7 (clone: B20.1, 127822, BioLegend), CD31-Alexa Fluor 594 (clone: 390, 102432, BioLegend), PDPN-BV421 (clone: 8.1.1, 127423, BioLegend), CR1/2-Alexa Fluor 700 (clone: 7E9, 123432, BioLegend), CD25-Pacific Blue (clone: PC61, 102022, BioLegend), Ly6C-PerCP/Cy5.5 (clone: AL-21, 560525, BD Biosciences), Ly6G-BUV661 (clone: 1A8, 741587, BD Biosciences), CD69-BUV563 (clone: H1.2F3, 741234, BD BioSciences), TCRVb5.1/5.2-FITC (clone: MR9-4, 553189, BD BioSciences). T-bet-PerCP/Cy5.5 (clone: 4B10, 644806, BioLegend), BCL6-BUV563 (clone: K112-91, 568073, BD Biosciences), IFN-gamma-BV711 (clone: XMG1.2, 505836, BioLegend), TNF-Alexa Fluor 488 (clone: MP6-XT22, 506313, BioLegend), GZMB-PerCP/eFluor710 (clone: NGZB, 46-8898-82, Thermo Scientific), IL-10-APC (clone: JESS-16E3, 561059, BD Biosciences), FOXP3-PE/eF610 (clone: FJK-16s, 61-5773-82, Thermo Scientific), CD103-Alexa Fluor 700 (clone: 2E7, 121442, BioLegend).
Immunohistochemistry: Armenian Hamster anti-mouse CD3 (clone: 145-2C11, 14-0031-82, Thermo Scientific), Rabbit anti-mouse CD20 (clone: SP32, MA5-16334, Thermo Scientific), Polyclonal goat anti-mouse CXCL13 (AF470, R&D systems), Polyclonal goat anti-mouse BAFF (AF2106, R&D systems), Chimeric recombinant rabbit anti-mouse CD35 (clone: 8C12, MA5-47853, Thermo Scientific), Rat anti-mouse FDC (clone: FDC-M1, 551320, BD Biosciences), Polyclonal rabbit anti-mouse BCL6 (PA5-27390, Thermo Scientific), Alexa Fluor 488 Rat anti-mouse CD20 (clone: SA275A11, 150432, BioLegend), eFluor660 rat anti-mouse B220 (clone: RA3-6B2, 50-0452-82, Thermo Scientific), Alexa Fluor 488 goat anti-armenian hamster IgG (127-545-160, Jackson Laboratory), Alexa Fluor 594 donkey anti-rabbit IgG (A32754, Thermo Scientific), Alexa Flour 647 donkey anti-rat IgG (A78947, Thermo Scientific), Alexa Fluor 647-conjugated Donkey anti-goad IgG (A32849, Thermo Scientific). Alexa Fluor 594 anti-mouse I-A/I-E (clone: M5/114.15.2, 107650, BioLegend), APC anti-mouse Thy1.2 (clone: QA20B09, 117104, BioLegend), Alexa Fluor 594 anti-mouse CD31 clone: 390, 102432, BioLegend), Rabbit polyclonal anti-mouse S1PR2 (21180-1AP, Proteintech), Alexa Fluor 647-conjugated rat anti-mouse PD-1 (clone: 29F.1A12, 135230, BioLegend), Alexa Fluor 594-conjugated rat anti-mouse CD4 (clone: GK1.5, 100446, BioLegend), Polyclonal anti-GFP (A10262, Thermo Scientific), Alexa Fluor 647-conjugated donkey anti-rabbit IgG (A32795TR, Thermo Scientific), Alexa Fluor 488-conjugated donkey anti-rabbit anti-IgG (A-21206, Thermo Scientific), coralite594-conjugated anti-mouse Thy1.2 (clone: 30-H12, CL594-65088, Thermo Scientific), Alexa Fluor 594-conjugated anti-mouse CD4 (clone: GK1.5, 100424, BioLegend), Rat anti-mouse Blimp1 (clone: 6D3, 14-5963-82, Thermo Scientific).
in vivo injections: anti-CD4 (clone: GK1.5, BE0003-1, BioXcell), anti-CD8a (clone: 2.43, BE0061, BioXcell), rat IgG2b isotype control (clone: LTF-2, BE0090, BioXcell), anti-CD40 (clone: FGK4.5, BE0016, BioXcell), rat IgG2a isotype control (clone: 2A3, BE0089, BioXcell), anti-CD40L (clone: MR-1, BE0017-1, BioXcell), polyclonal armenian hamster IgG control (BE0091, BioXcell).
Magnetic isolation: EasySep Mouse Naive CD4+ T cell isolation kit (19765, STEMCELL Technologies), Mouse B cell Isolation Kit (19854, STEMCELL Technologies). Germinal Center B cell (PNA) MicroBead Kit, mouse (130-110-479, Miltenyi Biotec).
ELISA: sandwich OVA ELISA kit (LS-F9540-1, LS Bio).

Validation

Each antibody was validated for the species (mouse) and application (immunohistochemistry, flow cytometry, fluorescence activated cell sorting, and in vivo experiments) by the correspondent manufacturers. All of antibodies were tested using control tissues.

Eukaryotic cell lines

Policy information about cell lines and Sex and Gender in Research

Cell line source(s)	Commercially unavailable GL261 was kindly provided by Dr. Gavin Dunn. CT2A cell line was purchased from Sigma-Aldrich (SCC194). HEL-OVA-expressing cells lines were generated in the laboratory.
Authentication	Cell lines have been checked for morphological features routinely. The short tandem repeat (STR) profiling has been done.
Mycoplasma contamination	All cell lines were confirmed to be free of Mycoplasma contamination by PCR.
Commonly misidentified lines (See ICLAC register)	No commonly misidentified cell lines are employed in this study.

Animals and other research organisms

Policy information about studies involving animals; ARRIVE guidelines recommended for reporting animal research, and Sex and Gender in Research

Laboratory animals	Specific pathogen-free (SPF) wild-type (WT) C57BL/6J (JAX:000664) mice were obtained from The Jackson Laboratory. The following strains were also purchased from The Jackson Laboratory: IL21-VFP (B6.Cg-Il21tm1.1Hm/DcrJ, JAX:03295), IL21R knockout (B6.129-Il21rtm1Kopf/J, JAX:019114), OT-II (B6.Cg-Tg(TcraTcrb)425Cbn/J, JAX:004194), MD4 (C57BL/6-Tg(IghelMD4)4Ccg/J, JAX:002595), Jchain-CreERT2 (B6(129)-Jchainem1(icre/ERT2)Deep/J, JAX:035764), ROSA-DTA (B6.129P2-Gt(ROSA)26Sortm1(DTA)Lky/J, JAX:009669), Ai9 (B6.Cg-Gt(ROSA)26Sortm9(CAG-tdTomato)Hze/J, JAX:007909), Thy1.1 (B6.PL-Thy1a/CyJ, JAX:000406), AID-Cre (B6.129P2-Aicdatm1(cre)Mnz/J, JAX:007770), Ai14 (B6.Cg-Gt(ROSA)26Sortm14(CAG-tdTomato)Hze/J, JAX:007914), UBC-GFP (C57BL/6-Tg(UBC-GFP)30Scha/J, JAX:004353), IFNg KO (B6.129S7-Ifngtm1Ts/J, JAX:002287), LTA KO (B6.129S2-Ltatm1Dch/J, JAX:002258), Aqp4 KO (B6(Cg)-Aqp4<tm1.1Tsna>, RIKEN: RBRC10053) and muMT (B6.129S2-Ighmtm1Cgn/J, JAX:002288) mice. All animals were housed in a temperature-controlled (22°C) and humidity-controlled (33-39%) environment with a 12-hour light/dark cycle and had ad libitum access to food and water.<br>Gnotobiotic mice were housed in plastic flexible film isolators in the gnotobiotic facility, also under a 12-hour light/dark cycle. These mice were weaned onto autoclaved food, with ad libitum access to food and water. Unless otherwise specified, eight-week-old male mice were used for experiments. All procedures were approved by the Institutional Animal Care and Use Committee of Washington University in St. Louis (Protocol 23-0145).
Wild animals	No wild animals involved in this study.
Reporting on sex	Experiments were performed on males. However, results have been confirmed using females.
Field-collected samples	No field-collected samples involved in this study.
Ethics oversight	All experiments were performed under the approval of the Institutional Animal Care and Use Committee at Washington University in St. Louis (#23-0145).

Note that full information on the approval of the study protocol must also be provided in the manuscript.

Plants

Seed stocks	N/A
Novel plant genotypes	N/A
Authentication	N/A

Flow Cytometry

Plots

Confirm that:

☒ The axis labels state the marker and fluorochrome used (e.g. CD4-FITC).

☒ The axis scales are clearly visible. Include numbers along axes only for bottom left plot of group (a 'group' is an analysis of identical markers).

☒ All plots are contour plots with outliers or pseudocolor plots.

☒ A numerical value for number of cells or percentage (with statistics) is provided.

Methodology

Sample preparation	Mice were euthanized using a CO_2 chamber and transcardially perfused with DPBS. Single-cell suspensions were prepared from each organ. Bones were harvested and cleaned to remove attached soft tissues, and the dura was peeled from the skull. Single-cell suspensions from bone marrow were obtained as previously described. Briefly, bones were mechanically dissociated by chopping with scissors in Roswell Park Memorial Institute (RPMI) 1640 medium (Gibco, 11875) containing 2% FBS.Dura, chopped lymph nodes, brain, and brain tumor tissues were digested with a solution containing 1 mg/mL collagenase VIII (Sigma-Aldrich, C2139) and 0.5 mg/mL DNase I (Thermo Scientific, EN0521) at 37°C for 30 minutes. Whole spleens or single-cell suspensions from various organs were passed through 70-µm strainers. Cell suspensions from brain and tumor tissues were centrifuged with 30% Percoll (Sigma-Aldrich, 17-0891-01) to remove myelin debris. Red blood cells were removed using ammonium-chloride-potassium (ACK) lysis buffer (Gibco, A1049201). Single cell suspensions in FACS buffer (DPBS with 2% FBS) were incubated with anti-CD16/32 (1:200 dilution) for Fc blockade, followed by surface staining with antibodies (1:200 dilution). Viability was marked by Zombie NIR fixable viability dye (BioLegend). For intracellular staining, cells were fixed and permeabilized using the FOXP3 staining kit (BioLegend) according to manufacturer's instructions. Then, cells were incubated with antibodies with 1:100 ~ 1:200 dilution. All samples were acquired on an Aurora spectral flow cytometer (Cytek).
Instrument	Aurora Spectral Flow Cytometer (Cytek)<br>Cytek SpectroFlo for data collection.<br>FlowJo v10.8.1 for data analysis.
Software	CYTEK SpectroFlo for data collection and FlowJo v.10.8.1 for data analysis.
Cell population abundance	Viability dyes were used for each individual experiment. Control tissues, FMO, or isotype control antibodies were used for gating. For sorting, small fraction of sorted cells were used to confirm its purity.
Gating strategy	Gating strategies are provided in Extended Data Figures.<br>Briefly:<br>CD4 T cells: Live/Singlet/CD45+/CD3+/CD4+.<br>Tfh cells: Live/Singlet/CD45+/CD3+CD4+/IL-21VFP+CXCR5+ or Live/Singlet/CD45+/CD3+CD4+/PD-1+CXCR5+ (This population was further assessed with CD40L, ICOS, BCL6.<br>CD8 T cells: Live/Singlet/CD45+/CD3+/CD8a+.<br>Activated CD4 or CD8 T cells : Live/Singlet/CD45+/CD3+/CD4+ or CD8+/CD44+.<br>B cells: Live/Singlet/CD45+/CD19+B220+.<br>Developing B cells: Live/Singlet/CD45+/CD19+B220+/IgD-IgM-.<br>Immature B cells: Live/Singlet/CD45+/CD19+B220+/IgD+IgM-.<br>Mature B cells: Live/Singlet/CD45+/CD19+B220+/IgD+IgM+.<br>GC-like or GC B cells (Live/Singlet/CD45+/CD19+B220+/IgD-/CD38-FAS+ or Live/Singlet/CD45+/CD19+B220+/IgD-CD38-/FAS +AID-Ai14+).<br>(Immunoglobulin expression was assessed by surface IgA/IgG staining).<br>ASCs: Live/Singlet/CD45+/CD3-IgD-/CD138+TACI+ or Live/Singlet/CD45+/Jchain-TdTomato+TACI+.<br>Plasmablast: Live/Singlet/CD45+/CD3-IgD-/CD138+TACI+/B220+.<br>Plasma cell: Live/Singlet/CD45+/CD3-IgD-/CD138+TACI+/B220-.<br>(Immunoglobulin expression was assessed by intracellular IgM/IgA/IgG staining).<br>OT-II CD4 T cells: Live/Singlet/CD45+/CD3+CD4+/Thy1.2+TCRVa2+ or Live/Singlet/CD45+/CD3+CD4+/TCRVa2+TCRVb5.1/5.2+.<br>MD4 B cells: Live/Singlet/CD45+/CD19+B220+/IgMa+ or Live/Singlet/CD45+/CD19+B220+/IgMa+UBC-GFP+.<br>Microglia: Live/Singlet/CD45+/CD45lowCD11b+.<br>CD11b-CD45hi: Live/Singlet/CD45+/CD45hiCD11b-.<br>CD11b+CD45hi: Live/Singlet/CD45+/CD45hiCD11b+.<br>Tumor infiltrating NK cell: Live/Singlet/CD45+/CD45hiCD11b-/NK1.1+CD3-.<br>Tumor infiltrating CD4 T cells: Live/Singlet/CD45+/CD45hiCD11b-/CD3+/CD8a-CD4+.<br>Tumor infiltrating CD8 T cells: Live/Singlet/CD45+/CD45hiCD11b-/CD3+/CD8a+CD4-.<br>Tumor infiltrating Tregs: Live/Singlet/CD45+/CD45hiCD11b-/CD3+/CD8a-CD4+/FOXP3+CD25+.<br>(Functions were assessed by PD-1, CD44, GZMB, IFNg, TNF, IL-10).<br>Monocyte: Live/Singlet/CD45+/CD45hiCD11b+/Ly6C+Ly6Glo/-.<br>Neutrophil: Live/Singlet/CD45+/CD45hiCD11b+/Ly6G+Ly6Clo/-.

☒ Tick this box to confirm that a figure exemplifying the gating strategy is provided in the Supplementary Information.
